# Construction of immune-related signature and identification of S100A14 determining immune-suppressive microenvironment in pancreatic cancer

**DOI:** 10.1186/s12885-022-09927-0

**Published:** 2022-08-11

**Authors:** Chengcheng Wang, Yuan Chen, Yin Xinpeng, Ruiyuan Xu, Jianlu Song, Rexiati Ruze, Qiang Xu, Yupei Zhao

**Affiliations:** Department of General Surgery, State Key Laboratory of Complex Severe and Rare Diseases, Peking Union Medical College Hospital, Chinese Academy of Medical Sciences, Peking Union Medical College, Beijing, 100023 PR China

**Keywords:** S100A14, Tumor immune microenvironment, CD8 + T cells, Prognostic signature, Immunotherapy, PD-L1, Pancreatic cancer

## Abstract

Pancreatic cancer (PC) is a highly lethal and aggressive disease with its incidence and mortality quite discouraging. A robust prognostic signature and novel biomarkers are urgently needed for accurate stratification of the patients and optimization of clinical decision-making. Since the critical role of immune microenvironment in the progression of PC, a prognostic signature based on seven immune-related genes was established, which was validated in The Cancer Genome Atlas (TCGA) training set, TCGA testing set, TCGA entire set and GSE71729 set. Furthermore, S100A14 (S100 Calcium Binding Protein A14) was identified as the gene occupying the most paramount position in risk signature. According to the GSEA, CIBERSORT and ESTIMATE algorithm, S100A14 was mainly associated with lower proportion of CD8 + T cells and higher proportion of M0 macrophages in PC tissue. Meanwhile, analysis of single-cell dataset CRA001160 revealed a significant negative correlation between S100A14 expression in PC cells and CD8 + T cell infiltration, which was further confirmed by tissue microenvironment landscape imaging and machine learning-based analysis in our own PUMCH cohort. Additionally, analysis of a pan-pancreatic cancer cell line illustrated that S100A14 might inhibit CD8 + T cell activation via the upregulation of PD-L1 expression in PC cells, which was also verified by the immunohistochemical results of PUMCH cohort. Finally, tumor mutation burden analysis and immunophenoscore algorithm revealed that patients with high S100A14 expression had a higher probability of responding to immunotherapy. In conclusion, our study established an efficient immune-related prediction model and identified the potential role of S100A14 in regulating the immune microenvironment and serving as a biomarker for immunotherapy efficacy prediction.

**Supplementary Information:**

The online version contains supplementary material available at 10.1186/s12885-022-09927-0.

## Introduction

Pancreatic cancer (PC) is one of the most lethal malignancies, with a five-year survival rate of only 11% in the United States [1]. The global burden of PC has increased dramatically over the past few decades and is expected to become the second most common cause of cancer-related mortality by 2030 after lung cancer [2, 3]. Beyond the scarcity of sensitive screening methods and the emergence of chemoresistance, this dismal situation is largely attributable to the lack of effective risk prediction signature and biomarkers [4], which hinders the individualized treatment of PC to some extent. Therefore, it is of great significance to establish validated prediction signature and screen potential novel biomarkers for the accurate assessment of patient’s prognosis and optimization of clinical decision-making.

Tumor immune microenvironment (TIME), defined as all immunological components within tumors, mainly comprises innate immune cells, adaptive immune cells, extracellular immune factors and cell surface molecules [5, 6]. Extensive studies have shown that tumor immune microenvironment possessed profound effect on tumor development [7, 8]. For example, IL-10 prevents dendritic cell-mediated CD8 + T cell apoptosis, thus potentiating CD8 + T cell-mediated antitumor immunity [9]. And transforming growth factor beta (TGFβ) functions as an immunosuppressive factor through inhibition of CXCR3 in CD8 + T cells, thereby limiting their trafficking into tumors [10]. Therefore, we consider a risk prediction signature based on immune-related genes (IRGs) to better predict the prognosis of PC and assist clinical decision-making. Furthermore, the most paramount gene in the risk signature was further explored, as well as its potential mechanisms and ability to serve as a novel biomarker for the efficiency of immunotherapy.

In the present study, we constructed a prediction signature of PC prognosis consisting of seven IRGs and the corresponding nomogram, which was validated in the training set, testing set, entire set and GSE71729 set respectively. Furthermore, S100A14 (S100 Calcium Binding Protein A14), a highly conserved elongation factor (EF)-hand calcium-binding protein, was identified as the gene occupying the most paramount position in the risk signature. GSEA, CIBERSORT and ESTIMATE algorithm suggested that S100A14 might be closely related to the PC immune microenvironment. Meanwhile, analysis of the single-cell dataset CRA001160 illustrated that CD8 + T cells might be the primary target of S100A14 function. Subsequently, tissue microenvironment landscape imaging and machine learning-based analysis were applied to visualize and analyze the immune microenvironment of PC patients in our independent cohort (PUMCH cohort). And the results showed a significant negative correlation between S100A14 expression and CD8 + T cell infiltration in tumor. In addition, a pan-pancreatic cancer cell analysis of CCLE database and iRegulon analysis suggested that S100A14-FOXL1-PD-L1 pathway may be a potential signal axes inhibiting the activation of CD8 + T cells, which was also verified by immunohistochemical results of PUMCH cohort. Finally, it was predicted that patients with high S100A14 expression had a higher probability of responding to anti-PD-1 and anti-CTLA-4 therapy, suggesting that S100A14 may serve as a potential biomarker for predicting PC immunotherapy efficiency.

## Materials and methods

### Datasets sources and processing

IRGs were extracted and integrated from the ImmPort database (https://immport.niaid.nih.gov; ≤ Mar 1, 2021) [11]. The RNA sequencing (RNA-seq) data, mutation profile and clinicopathological information of the patients were downloaded from The Cancer Genome Atlas (TCGA) database (https://portal.gdc.cancer.gov/; ≤ March 1, 2021) and the University of California Santa Cruz (UCSC) Xena website (https://xenabrowser.net/datapages/; ≤ March 1, 2021) (Table 1, detailed in Table S1). Patients with incomplete clinical information and follow-up period less than 30 days were excluded. Finally, 166 PC patients were included in the study. The gene expression data were normalized to transcripts per million (TPM) values and transformed to log2(TPM + 0.01) for further analysis unless otherwise noted.

**Table 1:** Clinical and pathologic information of training set, testing set and entire set

	**Training set**		**Testing set**		**Entire set**	
Character	NUMBER	%	NUMBER	%	NUMBER	%
**Age**						
**Median**	65		64.5		65	
**Range**	35–88		39–81		35–88	
**Os (m)**						
**Median**	15.5		15.9		15.6	
**Range**	1.0–76.2		3.2–91.4		1.0–91.4	
**Status**						
** Alive**	57	49.14	19	38.00	76	45.78
** Dead**	59	50.86	31	62.00	90	54.22
**Gender**						
** Male**	65	56.03	25	50.00	90	54.22
** Female**	51	43.97	25	50.00	76	45.78
**AJCC_Stage**						
** I**	12	10.35	6	12.00	18	10.84
** II**	102	87.93	39	78.00	141	84.94
** III**	1	0.86	2	4.00	3	1.81
** IV**	1	0.86	3	6.00	4	2.41
**Grade**						
** G1**	17	14.66	9	18.00	26	15.66
** G2**	63	54.31	28	56.00	91	54.82
** G3**	34	29.31	13	26.00	47	28.31
** G4**	2	1.72	0	0.00	2	1.21
**T stage**						
** T1**	4	3.45	2	4.00	6	3.61
** T2**	16	13.79	5	10.00	21	12.65
** T3**	95	81.90	41	82.00	136	81.93
** T4**	1	0.86	2	4.00	3	1.81
**N stage**						
** N0**	31	26.72	14	28.00	45	27.11
** N1**	84	72.42	34	68.00	118	71.08
** Nx**	1	0.86	2	4.00	3	1.81
**M stage**						
** M0**	59	50.86	17	34.00	76	45.78
** M1**	1	0.86	3	6.00	4	2.41
** MX**	56	48.28	30	60.00	86	51.81

In addition, GSE15471, GSE28735, GSE62165 and GSE71729 dataset were downloaded from Gene Expression Omnibus (GEO) (http://www.ncbi.nlm.nih.gov/geo/) [12–15], which were performed on GPL570, GPL6244, GPL13667 and GPL20769 platform. Expression values were calculated using the robust multi-array average (RMA) algorithm except GSE71729. The normalized expression matrix of microarray data can be directly download from the GEO dataset. Proteomic data from Clinical Proteomic from Tumor Analysis Consortium (CPTAC) were analyzed in the UALCAN database (http://ualcan.path.uab.edu/index.html) [16, 17].

Furthermore, the processed single-cell dataset CRA001160 (including over 57,000 cells from 24 primary PDAC samples and 11 normal samples) was downloaded from Tumor Immune Single-cell Hub (TISCH) database (http://tisch.comp-genomics.org/) [18, 19] (Table 2, detailed in Table S2). And the RNA-seq data and proteomics data of all pancreatic cell lines was extracted from the Cancer Cell Line Encyclopedia (CCLE) database (https://portals.broadinstitute.org/ccle) [20].

**Table 2:** Clinical and pathologic information of CRA001160 set and PUMCH cohort

	**CRA001160 Set**		**PUMCH COHORT**	
Character	Number	%	Number	%
**Age**				
**Median**	59		65.5	
**Range**	36–72		38–80	
**Gender**				
** Male**	11	45.83	17	44.74
** Female**	13	54.17	21	55.26
**AJCC_Stage**				
** I**	9	37.50	9	23.68
** II**	12	50.00	21	55.26
** III**	3	12.50	7	18.42
** IV**	0	0	1	2.64
**Diabetes**				
** Y**	10	41.67	19	50.00
** N**	14	58.33	19	50.00
**T stage**				
** T1**	4	16.67	5	13.16
** T2**	14	58.33	22	57.90
** T3**	5	20.83	9	23.68
** T4**	1	4.17	2	5.26
**N Stage**				
** N0**	10	41.67	15	39.47
** N1**	11	45.83	19	50.00
** N2**	3	12.5	4	10.53
**M Stage**				
** M0**	24	100.00	37	97.36
** M1**	0	0.00	1	2.64

### Establishment and validation of the prognostic signature based on IRGs

Limma package was applied to screen differentially expressed genes (DEGs) in GSE15471, GSE28735, GSE62165 and GSE71729 datasets respectively [21]. |Fold Change|> 1.5 and false discovery rate (FDR) < 0.05 were set as the cutoffs for the DEGs. The intersection of the four differential gene sets was then used for least absolute shrinkage and selection operator (LASSO) regression analysis and multivariate Cox regression analysis to obtain most effective prognostic model. According to the regression coefficient and the corresponding gene expression in the signature, the risk signature was established as follows: Risk score = (expr_gene1_ × Coef_gene1_) + (expr_gene2_ × Coef_gene2_) + … + (expr_genen_ × Coef_genen_). The Kaplan–Meier (KM) survival analysis, time-dependent receiver operating characteristic (ROC) curves and survival point diagram were utilized to evaluate the predictive efficiency of the risk signature. Meanwhile, univariate and multivariate Cox regression analysis were performed to determine the independence of the risk signature. Gene mutation status of the seven genes was obtained from the cBioportal database (http://www.cbioportal.org/) [22], and protein expression images in normal and tumor tissues was downloaded from the Human Protein Atlas (HPA) database (https://www.proteinatlas.org/).

In addition, the nomogram was also established to predict the 1-, 2-, 3-year survival probability based on the risk score and other clinicopathological characteristics. Univariate and multivariate Cox regression analysis were conducted to screen for valid clinicopathological factors included in the nomogram, and the corresponding time-dependent ROC curves, C-index calculation and calibration curves were applied to assess the efficiency of the nomogram.

### GSEA and iRegulon analysis for DEGs between S100A14 high and low expression group

Patients in the TCGA entire set were divided into S100A14 high expression group (*n* = 83) and S100A14 low expression group (*n* = 83) according to the median expression of S100A14. DEGs between the two groups were obtained by edgeR package in R language (|Log2FC|> 2 and FDR < 0.001), followed by GSEA analysis to determine the potential role of S100A14 in PC development [23]. The ALL ontology of the DEGs was analyzed by Gene Ontology (GO) [24], while pathway enrichment was analyzed by the Kyoto Encyclopedia of Genes and Genomes (KEGG) [25]. The number of random sample permutations was set at 1000, and NOM *p*-value < 0.05 and FDR q-value < 0.25 were set as the significance threshold. For transcription factors prediction, DEGs between S100A14 high and low expression group were analyzed by iRegulon tools in Cytoscape (version v3.7.1) [26].

### Estimation of tumor immune microenvironment

The ESTIMATE algorithm was able to estimate the content of mesenchymal and immune cells in PC tissue based on RNA-seq data [27], resulting in stromal score, immune score and estimate score. And the CIBERSORT algorithm was performed to quantify the relative abundance of 22 immune cells infiltrated in tumor microenvironment by a deconvolution algorithm [28]. These two algorithms were applied in the TCGA entire set and GSE71729 dataset respectively to evaluate the relationship between S100A14 expression and immune infiltration in the two datasets.

### Tumor mutation burden analysis

The mutation profile was acquired from TCGA data portal (https://portal.gdc.cancer.gov/; ≤ March 1, 2021). The landscape of somatic mutation in S100A14 low and high expression group was analyzed and visualized through “maftools” package in R language respectively [29]. Meanwhile, tumor mutation burden (TMB) and mutation frequency of top 10 genes were calculated and compared between S100A14 low and high expression groups.

### Prediction of the patients’ response to immunotherapy

Immunophenoscore (IPS), a scoring system for the quantification of tumor immunogenicity, has been verified to positively correlated to the responding rate to immunotherapy of PC patients [30]. The IPS data of PC patients was extracted from The Cancer Immunome Atlas (https://tcia.at/) for the following analysis, including the scores for anti-PD-1 therapy, anti-CTLA-4 therapy and the combination of the two therapies.

### Clinical specimens and immunohistochemical analysis

A total of 38 patients with primary PDAC who underwent surgical resection at Peking Union Medical College Hospital (PUMCH) were included in this study (PUMCH cohort, Apr. 2020-Nov. 2020). Informed consent was obtained from all patients, and this study was approved by the ethical committees of Peking Union Medical College Hospital. Manual staining was performed as the protocol previously described [31]. For primary antibody incubation of each patient, sections were incubated with rabbit monoclonal anti-PD-L1 antibody (1: 200) (Abcam, ab205921) for 1 h.

### Cell culture

All pancreatic cancer cell lines and pancreatic normal ductal cell line were purchased from the American Type Culture Collection (ATCC, Manassas, VA, USA). Meanwhile, all of the cells were authenticated by short tandem repeat (STR) analysis and regularly tested for mycoplasma contamination. BxPC-3 and HPAF-II cell lines were cultured in RPMI-1640 medium (Corning, #10–040-CV). CFPAC-1 and Capan-1 cell lines were cultured in Iscove’s Modified Dulbecco Medium (IMDM; Corning, #15–016-CV). PANC-1, MIA PaCa-2, SW1990, PaTu 8902, AsPC-1, T3M4 and Panc 10.05 cell lines were cultured in high glucose Dulbecco’s Modified Eagle Medium (DMEM; Corning, #10–013-CMR). All medium were supplemented with 10% fetal bovine serum (HyClone, #SH30073.03) and 1% Penicillin–Streptomycin (Life Technologies, #15,140–122). Cells were routinely maintained at 37℃ with 5% CO2.

### RNA isolation and qRT-PCR

The specific operation process has been described in the previous article [32]. All the values were normalized to GAPDH, and the 2-ΔCt method was used to quantify the fold change. The primer sequences used for qRT-PCR were as follows:

S100A14: Forward 5′- GAGACGCTGACCCCTTCTG-3’,

Reverse 5′- CTTGGCCGCTTCTCCAATCA-3’;

GAPDH: Forward 5′-GTCTCCTCTGACTTCAACAGCG-3’,

Reverse 5’-ACCACCCTGTTGCTGTAGCCAA-3’.

### Western blot analysis

Western blot analysis was performed as described previously [33]. The blot was cut prior to hybridisation with antibodies during blotting. Primary antibody for S100A14 was purchased from ProteinTech (10,489–1-AP, ProteinTech). And primary antibody for β-actin antibody was purchased from LabLead (A0101, LabLead).

### scRNA-seq data quality control, dimension reduction and cell clustering

The single-cell dataset CRA001160 was already processed by the author [19]. The Seurat package implemented in R language was applied to conduct following analysis. Low quality cells (< 200 genes/cell, < 3 cells/gene, > 5% mitochondrial genes, total expressed genes < 200 and total expressed genes > 7000) were removed. The gene expression profiles were then normalized and the top 2000 highly variable genes were generated to perform principal component analysis (PCA). Significant principal components were determined using JackStraw analysis and Elbow plot. PCs 1 to 15 were used for graph-based clustering (res = 1.0), which was visualized by the uniform manifold approximation and projection (UMAP) analysis. The cell types were annotated according to the markers provided by the author [19]. Meanwhile, T cells and macrophages were then extracted for further analysis.

### Tissue microenvironment landscape imaging and machine-learning based analysis

4 μm sections from the PUMCH cohort were used for tissue microenvironment landscape analysis through multiplex immunohistochemical kit (Panovue Biological Technology, 0,081,100,100). Sections were deparaffinized and tissues were fixed with 10% formalin, followed by antigen retrieval in heated EDTA buffer (pH 9.0, OriGene Technologies, ZLI-9069) for 15 min. Each section was put through three sequential rounds of staining, which includes blocking, primary antibody incubation, secondary horseradish peroxidase-conjugated polymer incubation and covalent binding of a different fluorophore using tyramide signal amplification. Between the two rounds, an additional antigen retrieval in heated EDTA Buffer (pH 9.0) for 15 min was conducted to remove bound antibodies. After all three sequential reactions, sections were counterstained with DAPI and mounted with antifade mounting medium. Slides were imaged and analyzed using the Vectra Multispectral Imaging System version 2 (Perkin Elmer) and the supporting software. Filter cubes used for multispectral imaging were DAPI, opal540, opal620, opal690. The corresponding imaging channels and antibody incubation are shown in Table S3.

### Statistical analysis

All statistical analysis were performed using R software (version 4.1.0) and GraphPad Prism 8 (version 8.0.1), including DEGs analysis, LASSO regression analysis, multivariate Cox regression analysis, clinicopathological factor analysis, K-M survival analysis, ROC curve analysis and correlation analysis. For qRT-PCR, data are means ± Standard Error of Mean (SEM) of three independent experiments. A two-sided *P* value < 0.05 was regarded to be statistically significant.

## Results

### Seven immune-related genes were screened out for constructing the risk signature

**Fig. 1:**
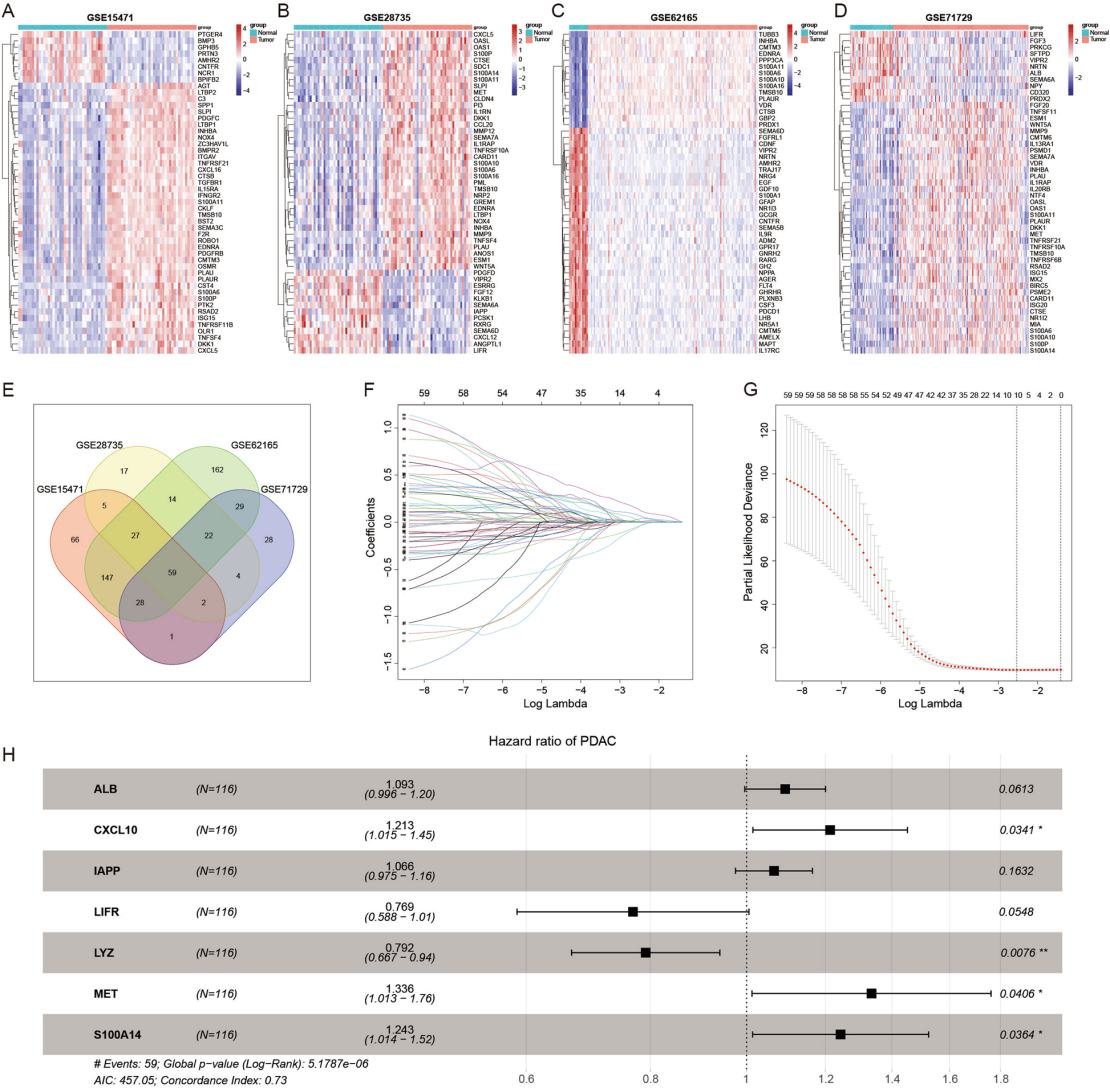
Screening out immune-related genes for risk signature construction. **A**-**D** Heatmap of immune-related DEGs between normal tissue and PC in GSE15471, GSE28735, GSE62165 and GSE71729. **E** Venn plot of the intersection of four DEGs datasets. **F** LASSO coefficient profiles of 59 prognostic IRGs. **G** Cross-validation for tuning parameter selection in the LASSO model. **H** Seven IRGs were screened out for constructing a risk signature

The process of the whole analysis was illustrated in Figure S1. A total of 1793 IRGs were integrated from the ImmPort database (Table S4). First, DEGs between normal and tumor samples were analyzed by limma package in GSE15471, GSE28735, GSE62165 and GSE71729 datasets. |Fold Change|> 1.5 and FDR < 0.05 were regarded as statistically significant, and 50 genes with the most significant differences in each dataset were shown in the heatmap (Fig. 1A-D).

By taking the intersection of these four differential gene sets, 59 common DEGs were obtained (Fig. 1E). Subsequently, LAASO regression analysis was applied to avoid overfitting problems and further screen the candidate genes (Fig. 1F-G, log(lambda.min) = -2.529272). And multivariate Cox analysis was then used to explore an appropriate gene combination for establishing the risk signature for PC patients. Finally, seven genes (ALB, CXCL10, IAPP, LIFR, LYZ, MET, S100A14) were screened out (Fig. 1H). Among them, LIFR and LYZ were protective factors for PC patients with Hazard Ratio (HR) < 1, and ALB, CXCL10, IAPP, MET and S100A14 were risk factors with HR > 1. Meanwhile, the mutation status and protein expression status of these genes were explored through the cBioportal database and HPA database (Figure S2-S3).

### Risk signature construction and evaluation for predicting survival rate of PC

Based on the gene expression and the regression coefficient derived from the multivariate Cox regression model, a risk-score formula was designed for PC patients’ survival prediction. The risk score for each patient was calculated as follows: Risk score = (0.0893 × expression level of ALB) + (0.1935 × expression level of CXCL10) + (0.0635 × expression level of IAPP) + (-0.2633 × expression level of LIFR) + (-0.2338 × expression level of LYZ) + (0.2893 × expression level of MET) + (0.2177 × expression level of S100A14). After that, each patient was assigned a risk score according to the above formula.

**Fig. 2:**
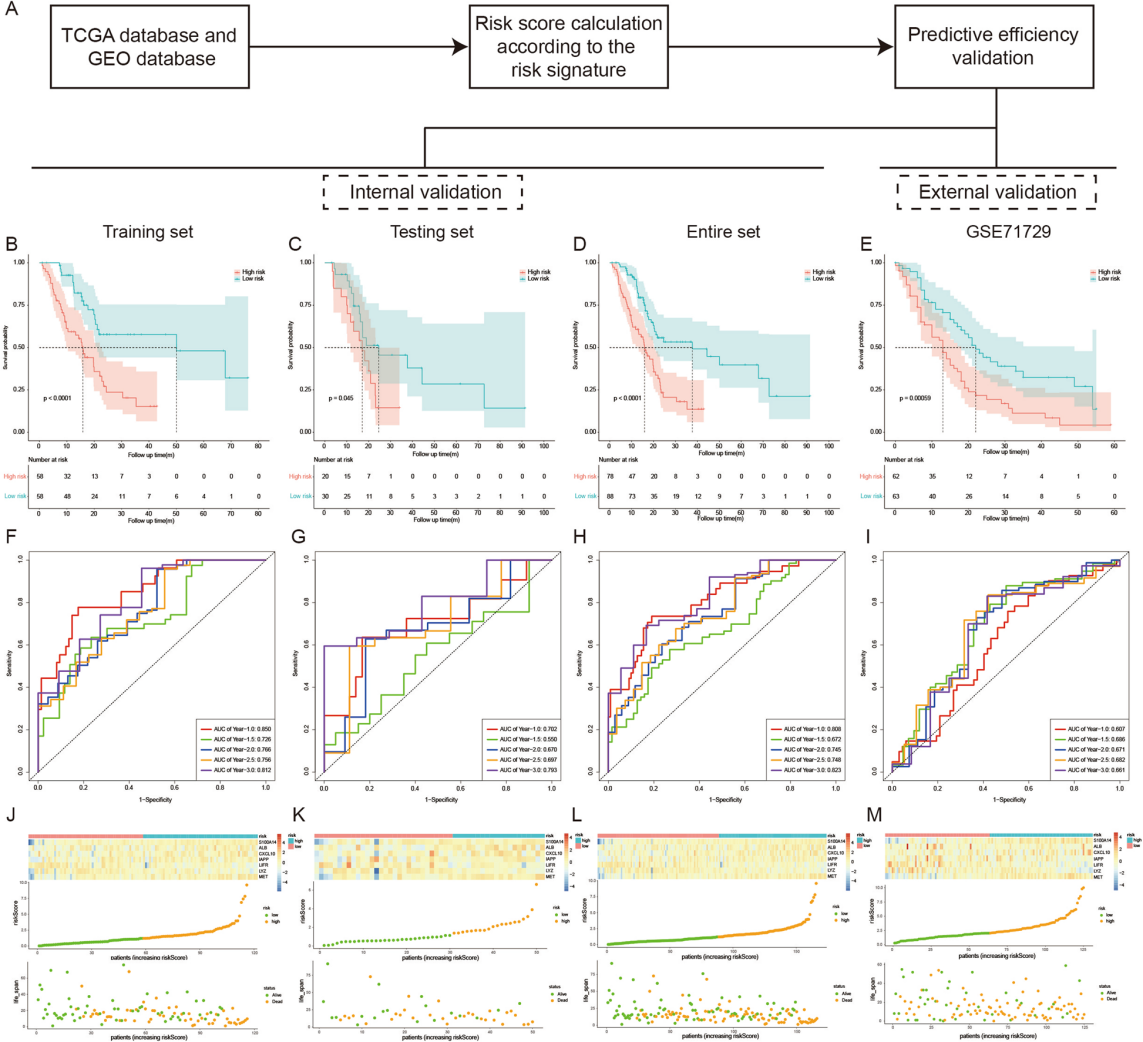
Effectiveness validation of the risk signature for survival prediction in training set, testing set, entire TCGA set and GSE71729 set. **A** The process of the risk signature validation. **B**-**E** Kaplan–Meier analysis of OS of the risk signature in training set, testing set, entire TCGA set and GSE71729 set. **F**-**I**) Time-dependent ROC analysis of the risk signature in the four datasets. **J**-**M** Heatmap of the nine hub genes expression, the risk scores distribution and survival status plots of the patients in the four datasets

In order to verify the validity of the model, we conducted internal validation in training set, testing set and entire set, as well as external validation in GSE71729 set (Fig. 2A). According to the Kaplan–Meier (K–M) curve analysis, patients in the high-risk group had significantly lower overall survival (OS) than those in the low-risk group in training set, testing set, entire set and GSE71729 set (Fig. 2B-E). At the same time, the area under curves (AUCs) of the risk signature for predicting 1-, 1.5-, 2-, 2.5-, and 3-year survival of PC patients were 0.850, 0.726, 0.766, 0.756, 0.812 in the training set, 0.702, 0.550, 0.670, 0.697, 0.793 in the testing set, 0.808,0.672, 0.745, 0.748, 0.823 in the entire set, and 0.607, 0.686, 0.671, 0.682, 0.661 in GSE71729 set respectively (Fig. 2F-I). These results demonstrated a high predictive efficacy of the signature in predicting the prognosis of PC. Meanwhile, compared with the low-risk group, the expressions of ALB, CXCL10, IAPP, MET and S100A14 increased in the high-risk group, whereas the expressions of LIFR and LYZ decreased. Consistently, the scatter plot of survival showed a gradual increase in the number of deaths as the risk score rose (Fig. 2J-M).

To further explore whether the predictive power of the signature was independent of other clinicopathological factors, univariate and multivariate Cox regression analysis was conducted in the training set, testing set, and entire set respectively. The results suggested an excellent independence of the risk signature, which was independent of gender, age, grade, AJCC_stage, T stage and N stage (Figure S4, *P* < 0.05 in all dataset for risk score). Meanwhile, the predictive efficacy of the signature was also tested in different subgroups stratified by gender, age, tumor grade and T stage. It was found that in almost all subgroups, patients in the high-risk group suffered significantly worse prognosis than those in the low-risk group (Figure S5, *P* < 0.05 in all subgroup except T1 + T2 group), further confirming the excellent independence of risk signature for PC prognosis prediction.

### Establishment and validation of a nomogram based on the seven-gene signature of PC

**Fig. 3:**
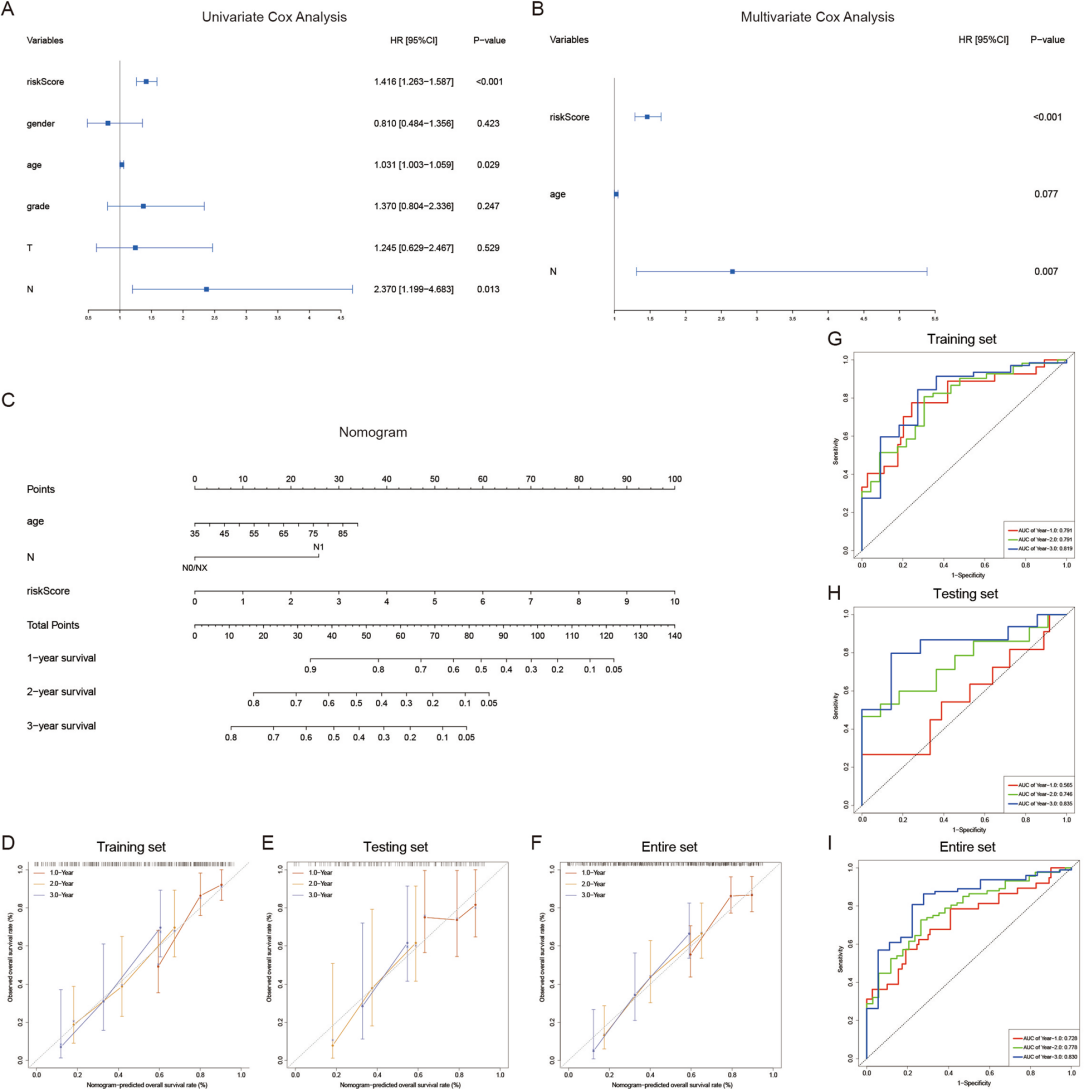
Nomogram construction for predicting 1-, 2- and 3-year survival rate of PC. **A**-**B** Univariate Cox regression analysis and multivariate Cox regression analysis in training set. **C** Nomogram integrating seven IRGs-based risk score, age and N stage. **D**-**F** The calibration plot of the nomogram for coherence test between 1-, 2- and 3-year OS prediction and actual outcome in the training set, testing set and entire set. **G**-**I** Time-dependent ROC analysis of the nomogram in training set, testing set and entire set

In order to optimize the prediction efficiency of the model, clinicopathological factors were also incorporated to construct a nomogram, including gender, age, grade, AJCC_stage, T stage and N stage. First, univariate Cox regression analysis was applied to preliminarily screen for various clinicopathological factors in training set, and factors with *P* < 0.2 were included in the multivariate Cox regression analysis (Fig. 3A-B). Concomitantly, we reconfirmed the independence of the risk signature in this process. Finally, risk score, age and N stages were incorporated into the establishment of nomogram for predicting 1-, 2-, and 3-year survival rate of PC. In the nomogram, the patients’ survival rates were estimated by the total points obtained by adding up the point of each factor (Fig. 3C). The C-index for the nomogram was 0.727, 0.603 and 0.689 in training set, testing set and entire set respectively, indicating that the nomogram possessed excellent predictive performance. Subsequently, the predictive power of the nomogram was further evaluated by calibration plot and time-dependent ROC curve. The calibration curves presented satisfied coherence between predicted and actual 1-year, 2-year and 3-year OS in training set, testing set and entire set (Fig. 3D-F). In addition, The AUCs of ROC curves for predicting 1-,2-, and 3-year survival were 0.791, 0.791, and 0.819 in the training set (Fig. 3G), 0.565, 0.746, and 0.835 in the testing set (Fig. 3H), and 0.728, 0.778, and 0.830 in the entire set, respectively (Fig. 3I).

### S100A14 was highly expressed and correlates with unfavorable prognosis in PC

**Fig. 4:**
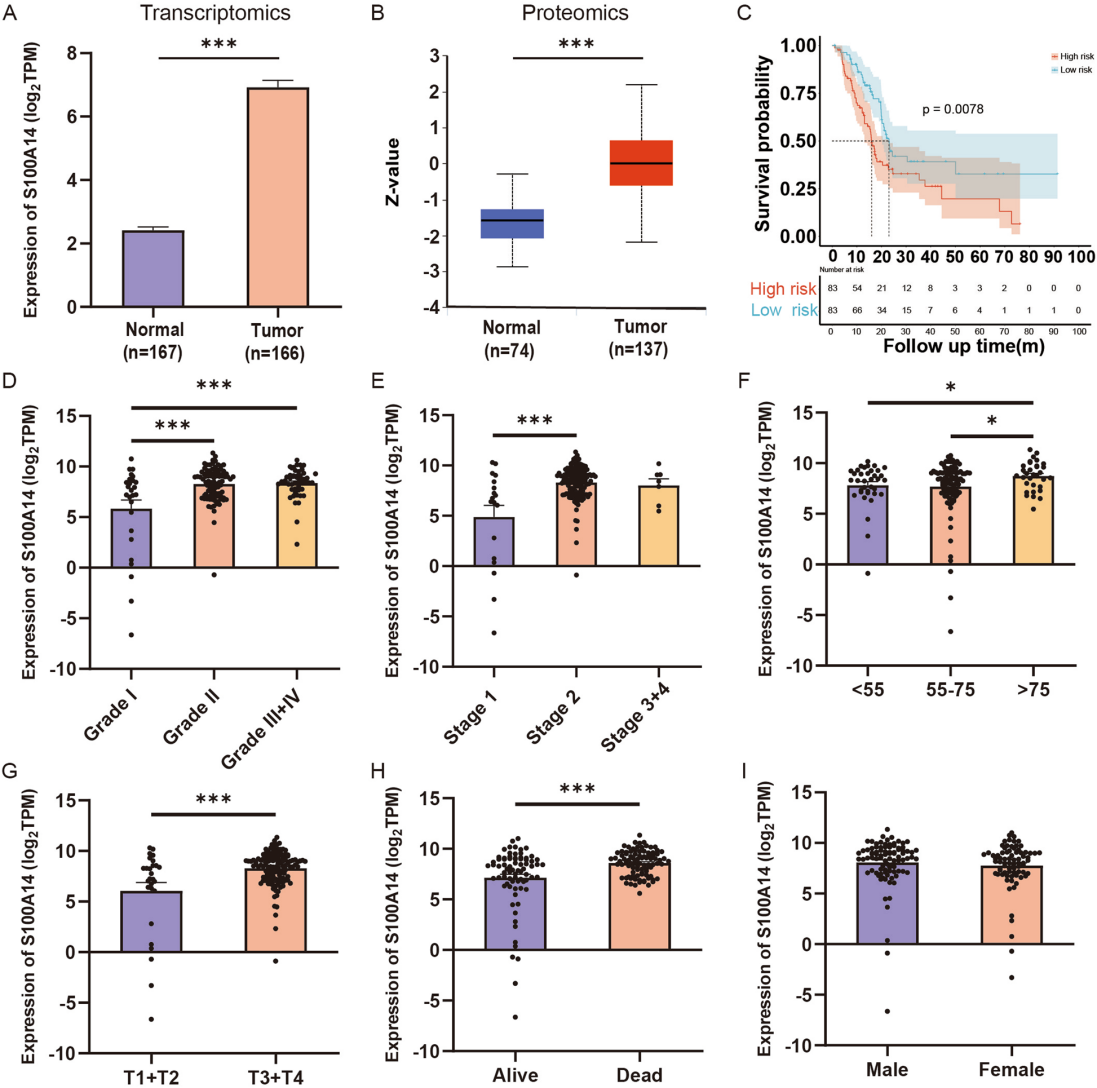
The correlation of the S100A14 expression and clinicopathological features of PC patients in TCGA entire set. **A** Expression difference of S100A14 between normal tissue and PC tissue according to RNA-seq data. **B** Expression difference of S100A14 between normal tissue and PC tissue according to proteomics data. **C** Kaplan–Meier analysis of OS between the high S100A14 expression group and low S100A14 expression group. **D**-**I** The correlation of S100A14 expression with tumor grade, AJCC_stage, age, T stage, N stage and status. *, *P* < 0.05; **, *P* < 0.01; ***, *P* < 0.001

Among the seven hub genes in the risk signature, S100A14 was the risk factor accompanied by the smallest *P* value. Furthermore, due to its high HR and coefficient in the risk signature, we tended to consider that S100A14 occupied the most paramount position in the risk signature. A joint analysis of TCGA and GTEx databases confirmed the remarkably high expression of S100A14 in tumor tissues (Fig. 4A). Meanwhile, the analysis from CPTAC database also indicated that S100A14 was significantly overexpressed in tumor tissue at the protein level (Fig. 4B). In addition, patients in the TCGA dataset were divided into high expression group (*n* = 83) and low expression group (*n* = 83) according to the median expression of S100A14. And the prognosis of patients in high expression group was significantly worse than that in low expression group (Fig. 4C). Concomitantly, the relationship between S100A14 expression and clinicopathological information of patients were further analyzed. Notably, the results revealed that the expression of S100A14 increased significantly with the progression of AJCC_stage, tumor grade, age and T stage (Fig. 4D-I).

### S100A14 predicts the infiltration of immune cells into PC microenvironment

**Fig. 5:**
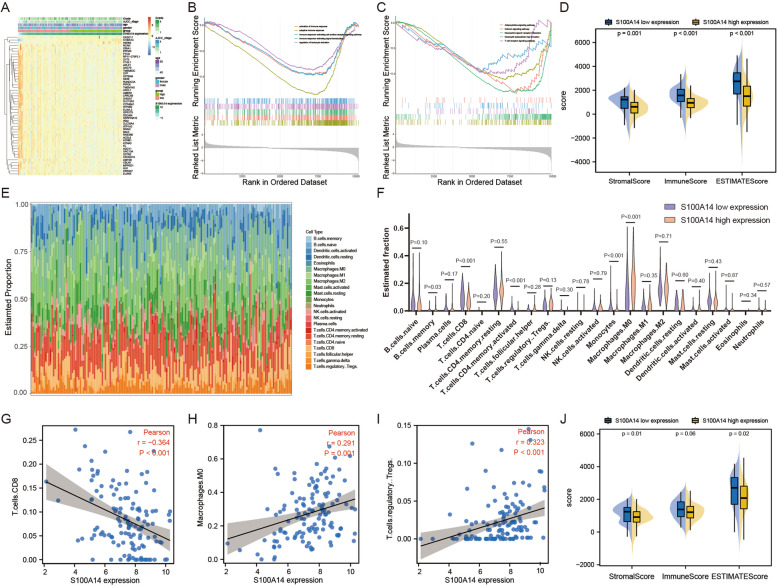
Immune cell infiltration difference between S100A14 high and low expression groups. **A** Heatmap of top 50 DEGs in PC between S100A14 high and low expression groups. **B**-**C** GSEA between S100A14 high and low expression groups. The representative 5 GO enrichments (**B**) and KEGG enrichments (**C**) were shown respectively. **D** Immunity score obtained by ESTIMATE algorithm in high and low S100A14 expression groups in TCGA dataset. **E**–**F** The abundance difference of the 22 types of immune cells between S100A14 high and low expression groups. **G**-**I** Correlation analysis between the S100A14 expression and the proportion of immune cells in GSE71729 dataset. Immune cell types with *P* < 0.05 were displayed. **H** Immunity score obtained by ESTIMATE algorithm in high and low S100A14 expression groups in GSE71729 dataset

Subsequently, in order to explore the in-depth mechanism of S100A14 leading to poor prognosis of PC, the patients in the TCGA entire set were divided into S100A14 high expression group (*n* = 83) and low expression group (*n* = 83). DEGs analysis was performed on the two groups and GSEA analysis was then conducted (Fig. 5A). Five representative pathways for the Gene Ontology (GO) and the Kyoto Encyclopedia of Genes and Genomes (KEGG) analysis were presented respectively (Fig. 5B-C). Overall, these pathways suggested that the function of S100A14 in pancreatic cancer may be related to the immunosuppressive tumor microenvironment in vivo.

Therefore, the ESTIMATE algorithm was applied to estimate the proportion of stromal cell and immune cell in PC patients, which revealed that a lower content of stromal cells and immune cells in the S100A14 high expression group (Fig. 5D). And the results of CIBERSORT, an algorithm which was designed to detect the proportions of 22 kinds of immune cells in tissues, revealed that relatively lower proportion of CD8 + T cells, activated memory CD4 + T cells and monocytes were found in the S100A14 high expression group compared with the low expression group. On the contrary, patients in S100A14 high expression group also possessed higher proportion of M0 macrophages cells and memory B cells (Fig. 5E-F). Moreover, in order to prove the universality of the results, the immune microenvironment of GSE71729 dataset (*n* = 125) was also analyzed. It was illustrated that the expression of S100A14 possessed a remarkable negative correlation with CD8 + T cells and significant positive correlation with M0 macrophages and Treg cells (Fig. 5G-I). In addition, the score for the tumor microenvironment by ESTIMATE package was similar in GSE71729 dataset (Fig. 5J), which further supported our hypothesis.

### Verification of the negative correlation between S100A14 expression and CD8 + T cells infiltration in PC

**Fig. 6:**
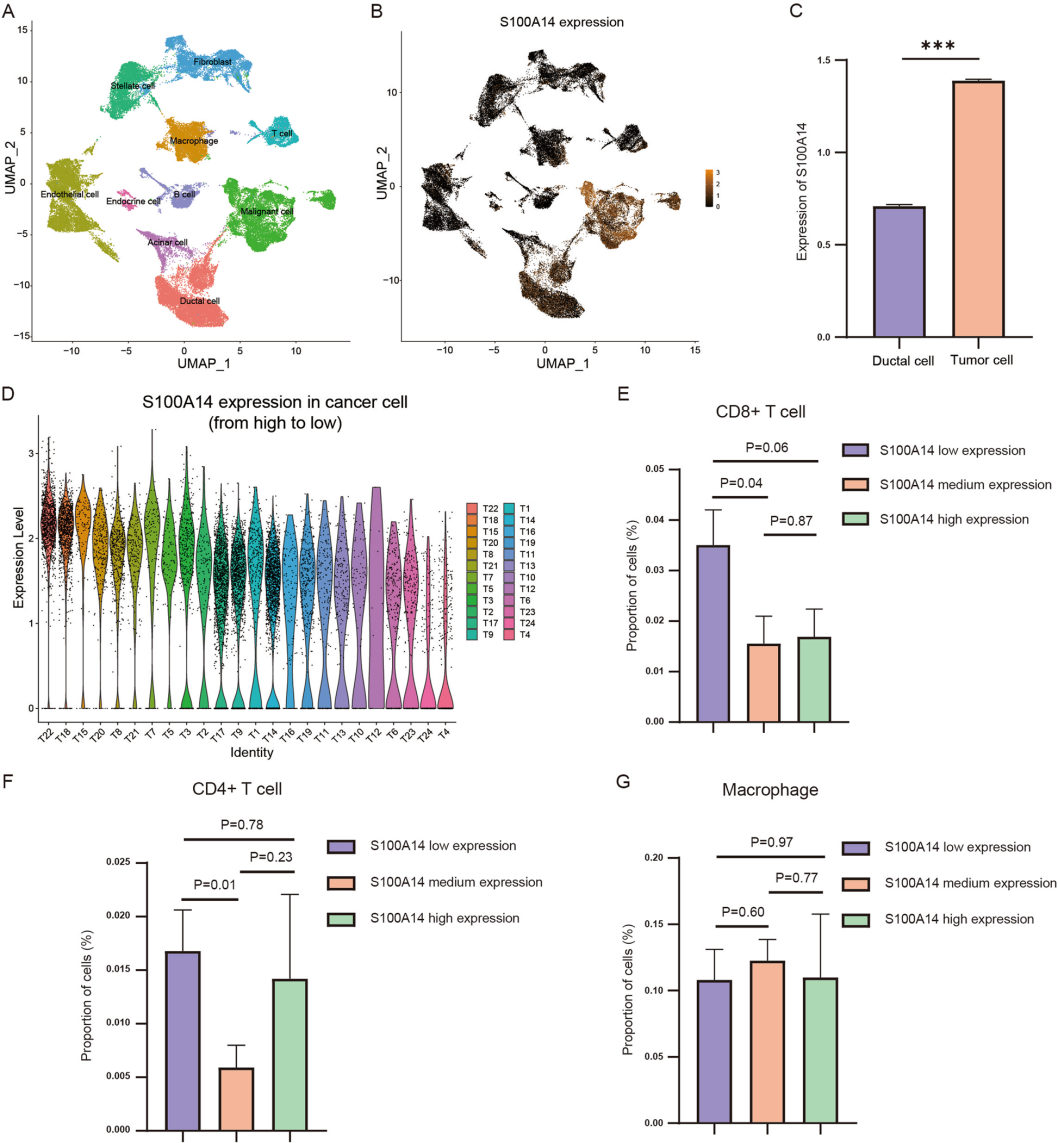
Analysis of S100A14 expression and immune cell infiltration in single-cell dataset CRA001160. **A** The UMAP plots of diverse cell types in PDAC tissues colored by major cell lineage. **B** Expression distribution of S100A14 in all cell types. **C** Comparison of S100A14 expression in normal ductal and PC cells. **D** Relative expression of S100A14 in cancer cells of each patient (ranked from high to low). **E**–**G** Comparison of infiltration of CD8 + T cells (**E**), CD4 + T cells (**F**) and macrophages (**G**) in S100A14 high, medium and low expression group. *, *P* < 0.05; **, *P* < 0.01; ***, *P* < 0.001

The algorithms above suggested that S100A14 was closely related to the immune microenvironment in PC, especially CD8 + T cells, CD4 + T cells and macrophages. Here, we further explored the effect of S100A14 on the immune microenvironment at the single-cell level. The single-cell dataset CRA001160 was analyzed in this process, which included 24 tumor samples and 11 normal samples. Firstly, dimensionality reduction clustering and expression distribution analysis of S100A14 showed that S100A14 was mainly expressed in tumor cells (Fig. 6A-B), and the expression of S100A14 in tumor cells was significantly higher than that in normal ductal cells (Fig. 6C). Subsequently, according to the S100A14 expression in tumor cells, patients were divided into S100A14 low expression group, S100A14 medium expression group and S100A14 high expression group (Fig. 6D). The number of CD8 + T cells, CD4 + T cells and macrophages were counted in each sample. Consistent with the previous results, with the increase of S100A14 expression, the proportion of CD8 + T cells gradually decreased (Fig. 6E). However, there was no significant trend in the relationship between the expression of S100A14 and the proportion of CD4 + T cells and macrophages (Fig. 6F-G).

**Fig. 7:**
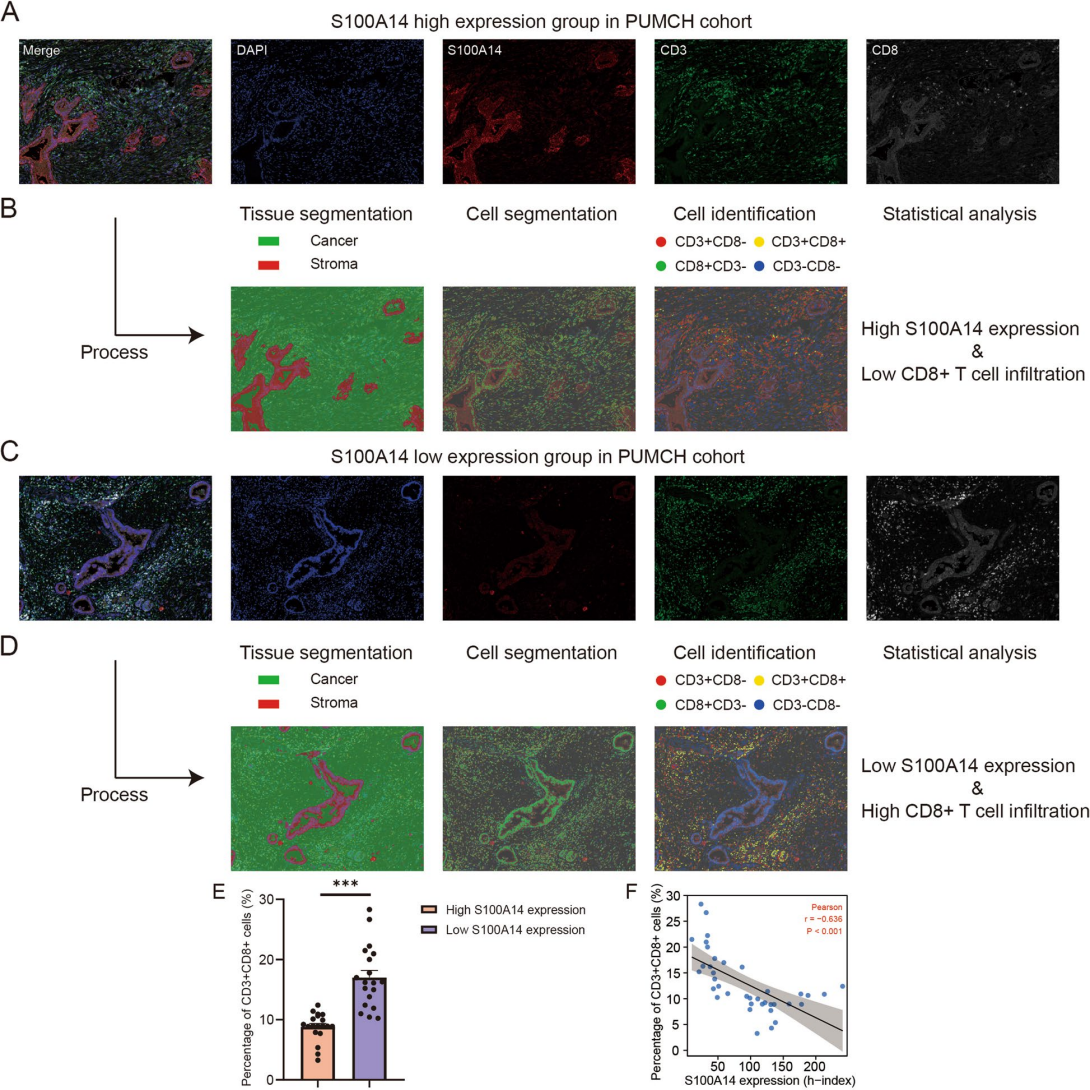
Tissue microenvironment landscape imaging and machine learning-based analysis in PUMCH cohort. **A**-**B** Representative images of samples with high S100A14 expression in PC cells and the process of tumor immune microenvironment analysis based on machine learning. **C**-**D** Representative images of samples with low S100A14 expression in PC cells and the process of tumor immune microenvironment analysis based on machine learning. **E** The proportion of CD3 + CD8 + T cells in stromal cells in S100A14 high and low expression group. **F** Correlation analysis of S100A14 expression in PC cells and proportion of CD8 + T cells in stromal cells. *, *P* < 0.05; **, *P* < 0.01; ***, *P* < 0.001

To further confirm this conclusion, tissue microenvironment landscape imaging and analysis were conducted on sections of 38 patients in our own PUMCH cohort, in which the association between the expression of S100A14 in tumor cells and the infiltration of CD8 + T cells was explored through machine learning. First, the tumor part and stroma part were separated by machine learning, and all the individual cells in the section were also separated simultaneously. Tumor cells were automatically scored for S100A14 expression, and cells in the stroma were also identified automatically through the membrane staining (CD3 + CD8 + cells were considered as CD8 + T cells). It was discovered that CD8 + T cell infiltration was extremely limited in tissues with high S100A14 expression. Conversely, CD8 + T cell infiltration was abundant in areas with low S100A14 expression (Fig. 7A-D). Consistently, statistical results also demonstrated that there was a significant negative correlation between the expression of S100A14 in tumor cells and the infiltration of CD8 + T cells in the stroma (Fig. 7E-F).

### The expression of S100A14 was positively correlated with PD-L1 in PC cells

**Fig. 8:**
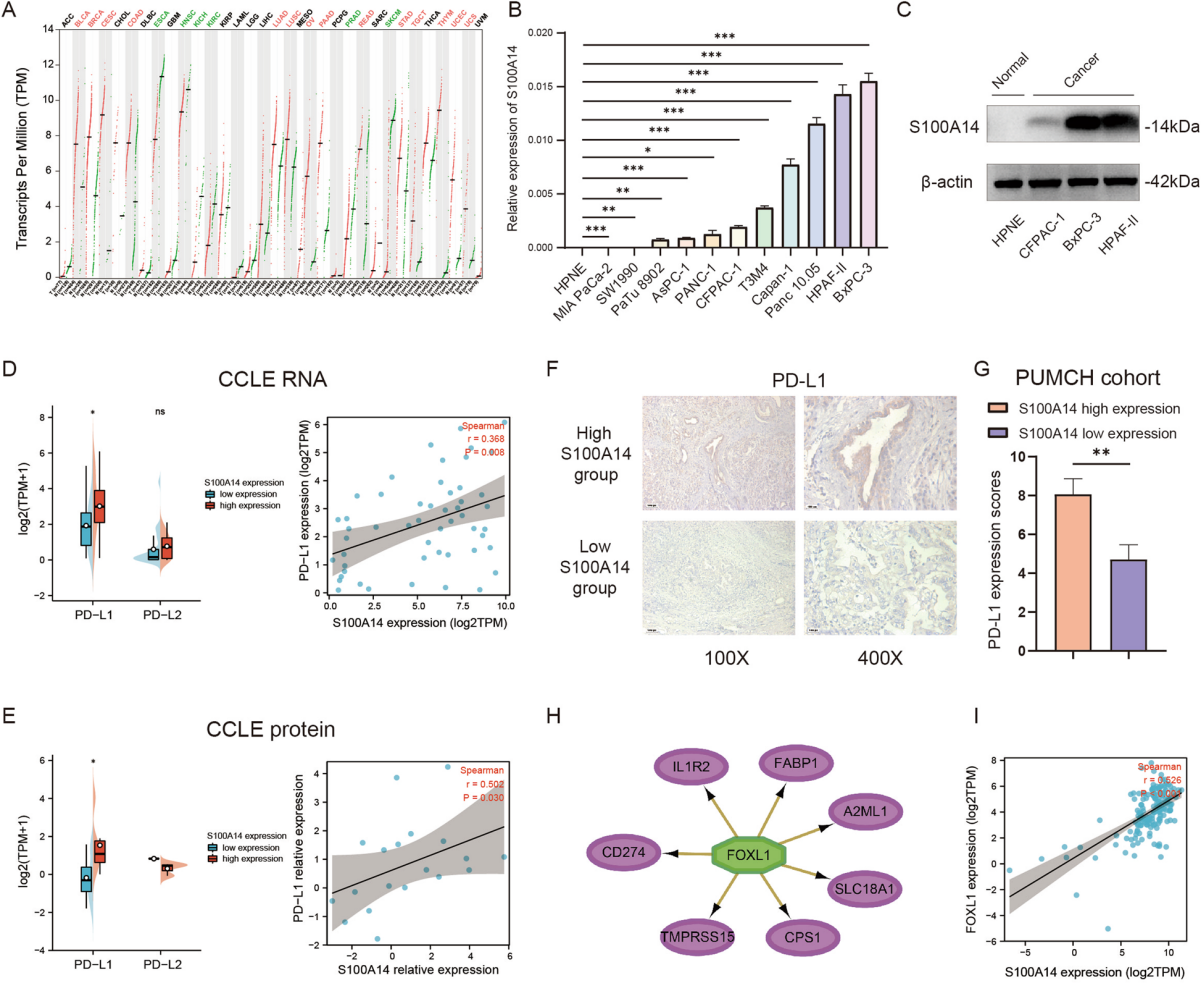
Expression of S100A14 in tumor cells and its relationship with PD-L1/2 expression. **A** A pan-cancer analysis of S100A14 on 33 types of tumors. Red represented a significant increase in tumor, green represented a significant decrease in tumor, and black meant no significant change. **B** Comparison of S100A14 expression between pancreatic normal ductal and PC cell lines detected by qRT-PCR. The difference between each PC cell line and HPNE was analyzed. **C** Comparison of S100A14 expression between pancreatic normal ductal and PC cell lines detected by western blot. **D** Relationship between S100A14 expression and PD-L1/2 expression at RNA level in PC cell lines. **E** Relationship between S100A14 expression and PD-L1 expression at protein level in PC cell lines. **F** Representative images of PD-L1 expression in S100A14 high and low expression group in PUMCH cohort. **G** Comparison of PD-L1 expression in S100A14 high and low expression group in PUMCH cohort. **H** Prediction of major downstream regulators caused by elevated S100A14 using iRegulon tools. **I** Relationship between S100A14 expression and FOXL1 expression

Since we have proved that S100A14 is associated with reduced infiltration of CD8 + T cells in the immune microenvironment of PC, the mechanism by which S100A14 lead to the deficiency of CD8 + T cells would be further explored. Firstly, a pan-cancer analysis of S100A14 revealed that PC experienced one of the most remarkably increase of S100A14 expression among all types of cancer (Fig. 8A), which was also confirmed by qRT-PCR and western blot in pancreatic normal ductal cell line and pancreatic cancer cell lines (Fig. 8B-C).

Subsequently, RNA-seq data and proteomics data of all pancreatic cancer cell lines were obtained from the CCLE database to explore whether the deficiency of CD8 + T cells was related to the high expression of immune checkpoints in PC cells. The results showed that the expression of S100A14 was significantly positively correlated with the expression of PD-L1, but not with the expression of PD-L2 at both RNA and protein levels (Fig. 8D-E). In addition, the results were also validated in our own PUMCH cohort, in which the PD-L1 immunohistochemical score of S100A14 high expression group was significantly higher than that of S100A14 low expression group (Fig. 8F-G).

Meanwhile, we further explored the potential mechanism of elevated PD-L1 expression in patients with high S100A14 expression. The gene co-expression network of patients with high S100A14 expression was analyzed by iRegulon in Cytoscape, which indicated that the transcription factor FOXL1 (Forkhead Box L1) may play a major regulatory role in the effect caused by high S100A14 expression (Fig. 8H). Moreover, the transcription factor was also predicted to regulate the expression of PD-L1 (Fig. 8H), and it was significantly positively correlated with S100A14 (Fig. 8I), suggesting that the S100A14-FOXL1-PD-L1 pathway may be one of the main signal axes regulating the immune desert microenvironment of PC induced by S100A14.

### S100A14 is associated with patients’ tumor mutation status and response to immunotherapy

**Fig. 9:**
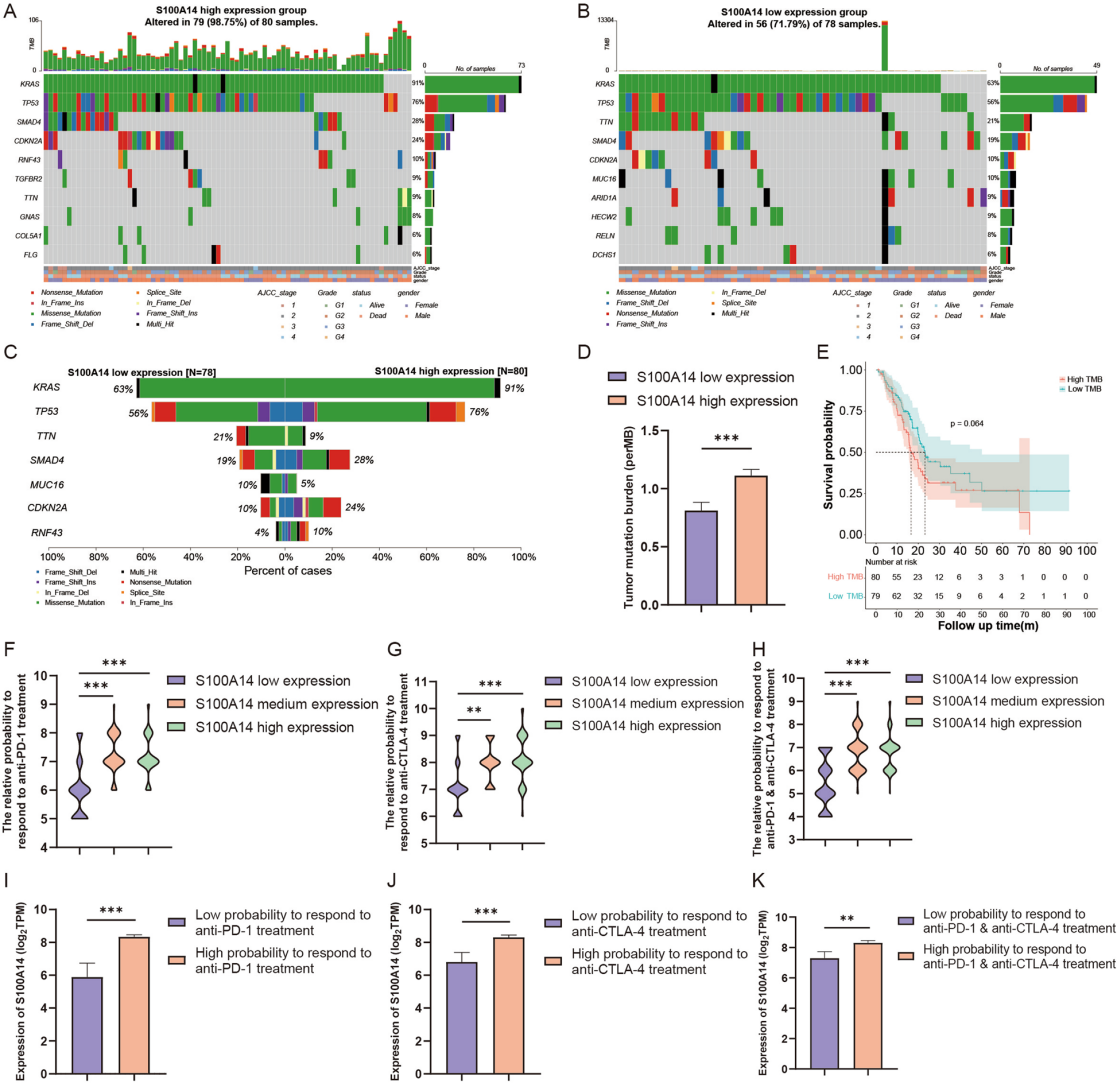
The mutation profile, TMB and relative probabilities of responding to immunotherapy in S100A14 high and low expression groups. **A**-**B** Mutation profile of PC patients in S100A14 high expression group and low expression group. **C** Mutation rate comparison of genes with high mutation rate between S100A14 high and low expression group. **D** The comparison of TMB between S100A14 high and low expression groups. **E** Kaplan–Meier analysis of OS between high and low TMB group. **F**-**K** The relationship between S100A14 expression and the relative probabilities of responding to immunotherapy, including anti-PD-1 therapy, anti-CTLA-4 therapy and the combination therapy. *, *P* < 0.05; **, *P* < 0.01; ***, *P* < 0.001

Mutation profiles of both the S100A14 high expression group and low expression group were analyzed and visualized (Figure S6-S7). For the S100A14 high expression group, the genes with the highest mutation rate were KRAS, TP53, SMAD4, CDKN2A, RNF43, TGFBR2, TTN, GNAS, COL5A1 and FLG. And for the S100A14 low expression group, the top 10 frequently mutation genes were KRAS, TP53, TTN, SMAD4, CDKN2A, MUC16, ARID1A, HECW2, RELN and DCHS1. It's worth noting that although KRAS and TP53 were genes with the highest mutation rate in both groups, the mutation rate was significantly different between the two groups (Fig. 9C, 91%: 63% for KRAS, 76%: 56% for TP53). Therefore, we further calculated the TMB for each patient and found that TMB in S100A14 high expression group was significantly higher than that in S100A14 low expression group (Fig. 9D), although TMB was not significantly associated with patients’ prognosis (Fig. 9E).

IPS, a machine learning-based scoring system, was able to predict the efficiency of immunotherapy, including anti-PD1 therapy, anti-CTLA4 therapy and a combination of the two therapies. According to the comprehensive analysis of S100A14 expression and IPS score, patients with high S100A14 expression possessed a relatively higher probability of responding to anti-PD1 therapy, anti-CTLA4 therapy and the combination of anti-PD1 and anti-CTLA-4 therapy (Fig. 9F-K). Therefore, S100A14 is expected to be a predictor of the efficacy of immunotherapy in PC patients, which is consistent with the previous conclusion that S100A14 might contribute to the progression of PC by promoting immune-suppressive tumor microenvironment.

## Discussion

PC is one of the most aggressive malignancies, which is expected to become the second leading cause of cancer-related death by 2030 [3]. The reasons for this situation are various, and the vital one is the lack of reliable predictive models and biomarkers, which hinders individualized treatment of PC. Herein, since the critical role of tumor immune microenvironment has been gradually revealed in recent years [5, 34], an IRGs-based predictive model was established to accurately assess the prognosis of PC patients, which is also verified in its validity and independence from different dimensions. Among the seven genes in the risk signature, CXCL10, IAPP, LIFR and MET have been reported to be involved in the carcinogenesis and progression of PC [35–38], which also demonstrates the considerable prognostic value of the risk signature to some extent. However, there has been limited coverage of ALB, LYZ and S100A14 in the field of PC, especially in the tumor immune microenvironment. Since S100A14 was the risk factor with the lowest *P* value, and S100A14 possessed relatively large HR and accounted for a high proportion in the risk signature, it was considered that S100A14 might occupy the core position in the risk signature. Therefore, S100A14 was paid special attention in the following analysis.

S100A14, an important member of S100 family proteins, is able to modulate biological process by functioning both as intracellular and extracellular factors [39, 40]. Meanwhile, S100A14 has been reported to be differentially expressed in various human malignancies and implicated in tumorigenesis and tumor progression [41, 42]. Notably, the effects of S100A14 are dual to some extent, depending on different biological processes and tumor types. Li et al. have reported that S100A14 could promote breast cancer metastasis by increasing the expression and secretion of CCL2/CXCL5 via RAGE-NF-κB pathway [43]. Conversely, another study conducted by Meng et al. identified S100A14 as a functional regulator suppressing nasopharyngeal carcinoma metastasis by inhibition of the NF-kB signaling pathway [44]. Here, the results of our study supported its deteriorating role during PC progression. To be specific, in the field of PC, studies have shown that S100A14 was able to promote proliferation, invasion, migration and gemcitabine resistance of PC cells [45]. However, the role of S100A14 in PC immune microenvironment has been limitedly reported, which is also the main content of this manuscript.

GSEA analysis illustrated that the high expression of S100A14 was associated to the weakened immune response in vivo. Therefore, CIBERSORT and ESTIMATE algorithm was further applied to reveal the proportion of various immune cells in PC patients' tissues. Patients with high S100A14 expression had significantly reduced CD8 + T cells, activated memory CD4 + T cells and monocytes, as well as increased M0 macrophages cells and memory B cells. In addition, the results of CIBERSORT algorithm in GSE71729 dataset also showed a similar trend. Based on the results of the two datasets, S100A14 may be associated with reduced CD8 + T cells and increased M0 macrophages in the immune microenvironment of PC. Subsequently, analysis of single-cell dataset CRA001160 revealed that S100A14 was mainly expressed in tumor cells. In addition, CD4 + T cells, CD8 + T cells and macrophages from each patient were extracted in from the single-cell dataset CRA001160. And the percentage of each immune cells to all stromal cells in each patient was then calculated. As a result, CD8 + T cells were significantly reduced in patients with high S100A14 expression, while there were no significant changes in CD4 + T cells and macrophages. According to the above results, the effect of high expression of S100A14 in tumor cells on the immune microenvironment of PC was finally focused on CD8 + T cells, the immune cell possessing the most prominent tumor killing ability [46, 47].

Tissue microenvironment landscape imaging system is a technology favoring the visualization and analysis of the tumor microenvironment through machine learning, which is able to automatically divide the tumor part and the stroma part, and identify the stroma cell type through the immunofluorescence of the cells. Here, we conducted tissue microenvironment landscape imaging in our PUMCH cohort (*n* = 38) to further explore the relationship between S100A14 expression and CD8 + T cell infiltration. The machine was designed to automatically segment the tumor cells and score the expression of S100A14 on the tumor cells. Subsequently, for stromal cells, CD3 + CD8 + cells were recognized as CD8 + T cells and their proportion in stromal cells was counted. Consistent with bioinformatics analysis, patients with high S100A14 expression had a lower proportion of CD8 + T cells in their cancer tissues, further confirming the above results from another dimension.

Afterwards, we aimed to explore how S100A14 leads to the deficiency of CD8 + T cells. Since the results above showed that S100A14 was mainly expressed in tumor cells, and previous studies had proved that S100 family proteins might up-regulate the expression of PD-L1 [32, 48], we hypothesized that S100A14 might be related to the dysregulation of immune checkpoints including PD-L1 and PD-L2, which had been widely acknowledged to inhibit T cell activation through activation of PD-1 receptors [49–51]. Therefore, the RNA-seq and proteomics data of all pancreatic cancer cell lines in the CCLE database were integrated and analyzed, which proved the expression of S100A14 was significantly positively correlated with the expression of PD-L1 in PC cell lines. Therefore, it was suggested that S100A14 might inhibit proliferation of CD8 + T cells and promote apoptosis of CD8 + T cells by up-regulating PD-L1 expression in PC cells.

Cancer immunotherapies, which manipulate the immune system to recognize and attack cancer cells, have become a powerful clinical strategy for treating cancer [52, 53]. However, immunotherapy has shown very limited efficacy in PC [54, 55]. Promisingly, a small subset of patients who exhibited high effector T-cell infiltration in tumor had longer overall survival [56, 57], suggesting that immunotherapy is still a potential treatment strategy for PC patients. And what we need to do is to identify patients with a high probability of responding to immunotherapy or explore optimal drug combinations to improve the effectiveness of immunotherapy.

Since we had explored the role of S100A14 in the immune microenvironment of PC, we wondered whether S100A14 might be a promising biomarker for predicting the immunotherapy response of PC patients. It has been widely reported that patients with high TMB had a relatively higher probability of responding to immunotherapy [58, 59]. Therefore, we explored the relationship between S100A14 expression and TMB, which suggested that patients with high S100A14 expression possessed a higher TMB, indicating a higher response rate to immunotherapy in this group. Consistently, the application of IPS algorithm also reached similar conclusion, indicating that S100A14 could be used as a potential biomarker for predicting the efficacy of immunotherapy in PC patients.

In spite of the positive results, the limitation of current study should be addressed as well. First of all, IPS is an algorithm based on machine learning to simulate the immunotherapy response of PC patients. Although its effectiveness has been widely verified in multiple datasets, it is still different from the real clinical situation to some extent. Secondly, due to the extremely poor prognosis of PC, there are few patients in the cohort with more than three years of survival, which may affect the long-term prediction efficiency of the risk signature.

## Conclusion

In summary, in this study, an IRGs-based prediction signature was constructed and validated in the training set, testing set, entire set and GSE71729 set respectively. Meanwhile, a nomogram was also established to further improve the prediction efficiency of the model. Subsequently, S10014 was identified as the gene occupying the most paramount position in this signature, which was proved to be increased significantly with the progression of AJCC_stage, tumor grade, age and T stage. GSEA, ESTIMATE and CIBERSORT algorithms demonstrated that the deteriorating effect of S100A14 on PC was mainly related to the dysregulation of immune microenvironment. Exploration of the single-cell dataset CRA001160 further demonstrated that high expression of S100A14 was associated with reduced infiltration of CD8 + T cells in tumor tissue. Subsequently, tissue microenvironment landscape imaging and machine learning-based immune microenvironment analysis were conducted in our PUMCH cohort, which confirmed the negative correlation between S100A14 and CD8 + T cell infiltration from another dimension. In addition, a pan-pancreatic cancer cell lines analysis, iRegulon analysis and immunohistochemical results of PUMCH cohorts suggested that S100A14-FOXL1-PD-L1 pathway may be a potential signal axes inhibiting CD8 + T cell activation. Finally, TMB analysis and IPS algorithm implied that S100A14 may serve as a potential biomarker for predicting the efficacy of immunotherapy in PC.

## Supplementary Information

**Additional file 1: Figure S1.** Flowchart of the whole study. **Figure S2.** The mutation status of the seven hub IRGs in TCGA dataset (A) and ICGC dataset (B). **Figure S3.** Representative images of hub IRGs expression status except CXCL10 and LIFR in normal pancreas and PC tissues. (The expression information of CXCL10 and LIFR was unavailable in HPA database). **Figure S4.** Independence of the risk signature and the other clinical variables, including, age, tumor grade, AJCC_stage and gender. (A, C, E) Univariate Cox regression analyses in the training set, testing set and entire set. (B, D, F) Multivariate Cox regression analyses in the training set testing set and entire set. **Figure S5.** Stratification analyses of all patients using the risk signature. (A-C) The Kaplan-Meier analysis of the younger stratum (age ≤ 65, n=88), older stratum (age >65, n=78) and all patients with PC (n=166). (D-F) The Kaplan-Meier analysis of the male stratum (n=90), female stratum n=76) and all patients with PC (n=166). (G-I) The Kaplan-Meier analysis of the Grade I/II stratum (n=117), Grade III/IV stratum (n=49) and all patients with PC (n=166). (J-L) The Kaplan-Meier analysis of the T1+T2 stratum (n=27), T3+T4 stratum (n=139) and all patients with PC (n=166). **Figure S6.** The mutation profiles of patients in S100A14 high expression group. **Figure S7.** The mutation profiles of patients in S100A14 low expression group.** Figure S8.** Correlation analysis of S100A14 and CD3E in the Bailey P. et al. cohort.

**Additional file 2. **

**Additional file 3: Table S1.** Detailed clinical and pathologic information of TCGA entire set. **Table S2.** Detailed clinical and pathologic information of CRA001160 dataset. **Table S3.** The imaging channels for each antibody and antibody incubation information in tissue microenvironment landscape imaging. **Table S4.** Immune-related genes obtained from the ImmPort database.

## Data Availability

The datasets used and/or analysed during the current study are available from the corresponding author on reasonable request.

## Abbreviations

PC: Pancreatic Cancer
S100A14: S100 Calcium Binding Protein A14
TIME: Tumor Immune Microenvironment
IRGs: Immune-related Genes
TMB: Tumor Mutation Burden
IPS: Immunophenoscore
GSEA: Gene Set Enrichment Analysis
