# Supplementary material for: Construction of immune-related signature and identification of S100A14 determining immune-suppressive microenvironment in pancreatic cancer
Source: BMC Cancer. 2022 Aug 11;22:879. doi: 10.1186/s12885-022-09927-0 (PMC9367131; doi:10.1186/s12885-022-09927-0)
Supplement: Supplementary file 1 — Additional file 1: Figure S1. Flowchart of the whole study. Figure S2. The mutation status of the seven hub IRGs in TCGA dataset (A) and ICGC dataset (B). Figure S3. Representative images of hub IRGs expression status except CXCL10 and LIFR in normal pancreas and PC tissues. (The expression information of CXCL10 and LIFR was unavailable in HPA database). Figure S4. Independence of the risk signature and the other clinical variables, including, age, tumor grade, AJCC_stage and gender. (A, C, E) Univariate Cox regression analyses in the training set, testing set and entire set. (B, D, F) Multivariate Cox regression analyses in the training set testing set and entire set. Figure S5. Stratification analyses of all patients using the risk signature. (A-C) The Kaplan-Meier analysis of the younger stratum (age ≤ 65, n=88), older stratum (age >65, n=78) and all patients with PC (n=166). (D-F) The Kaplan-Meier analysis of the male stratum (n=90), female stratum n=76) and all patients with PC (n=166). (G-I) The Kaplan-Meier analysis of the Grade I/II stratum (n=117), Grade III/IV stratum (n=49) and all patients with PC (n=166). (J-L) The Kaplan-Meier analysis of the T1+T2 stratum (n=27), T3+T4 stratum (n=139) and all patients with PC (n=166). Figure S6. The mutation profiles of patients in S100A14 high expression group. Figure S7. The mutation profiles of patients in S100A14 low expression group. Figure S8. Correlation analysis of S100A14 and CD3E in the Bailey P. et al. cohort. [file 12885_2022_9927_MOESM1_ESM.pdf]

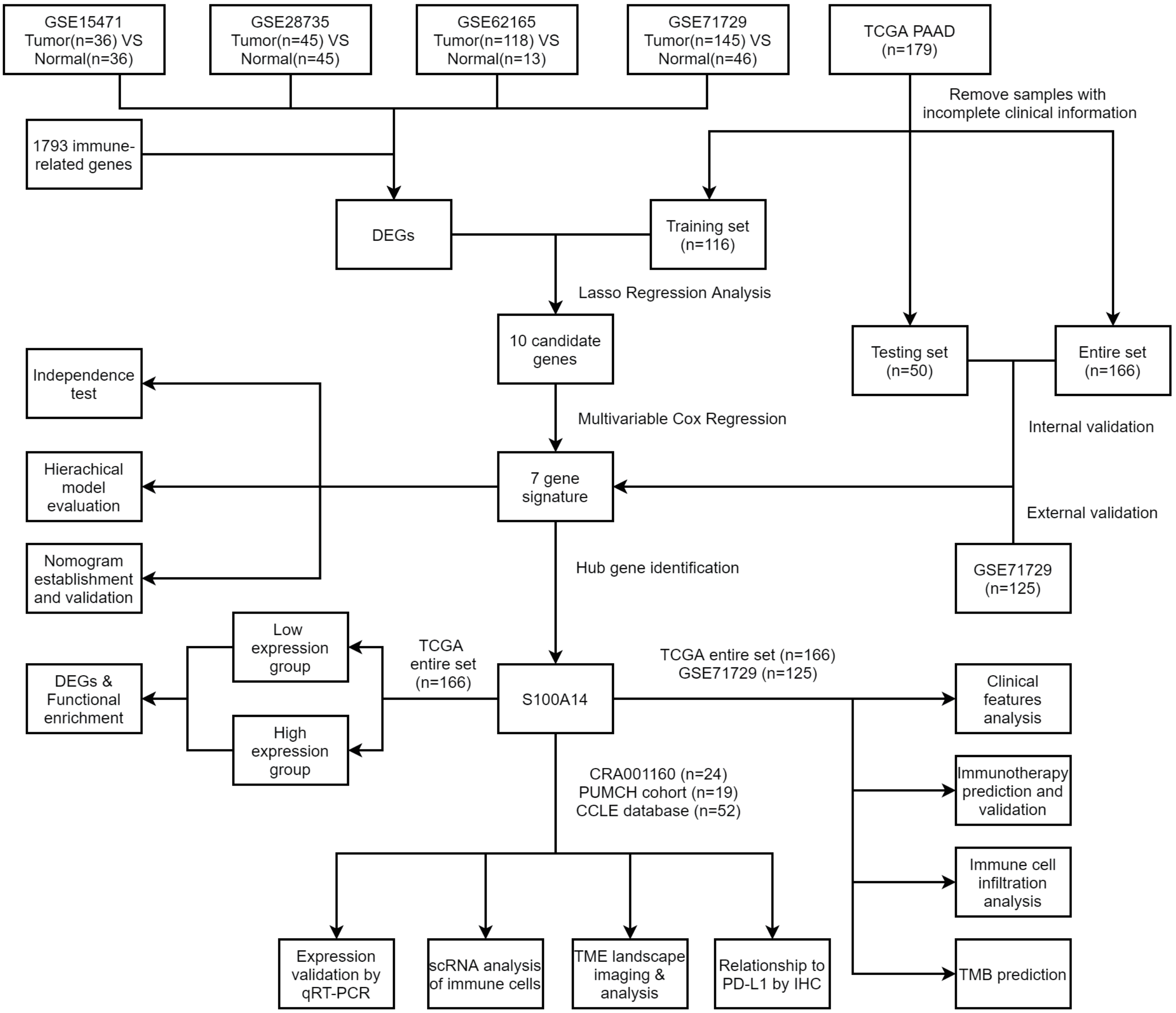

Figure S1. Flowchart of the whole study.

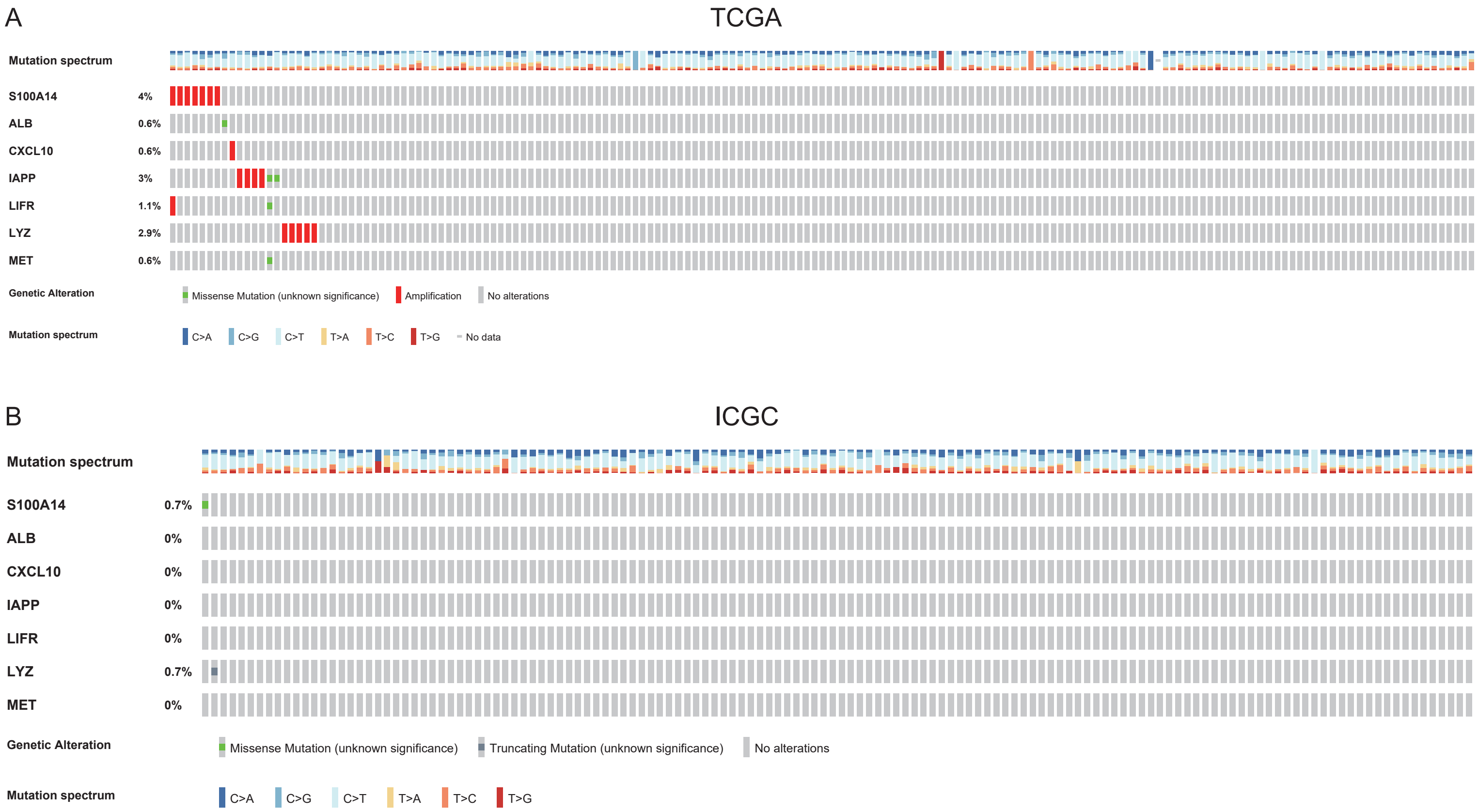

Figure S2. The mutation status of the seven hub IRGs in TCGA dataset (A) and ICGC dataset (B).

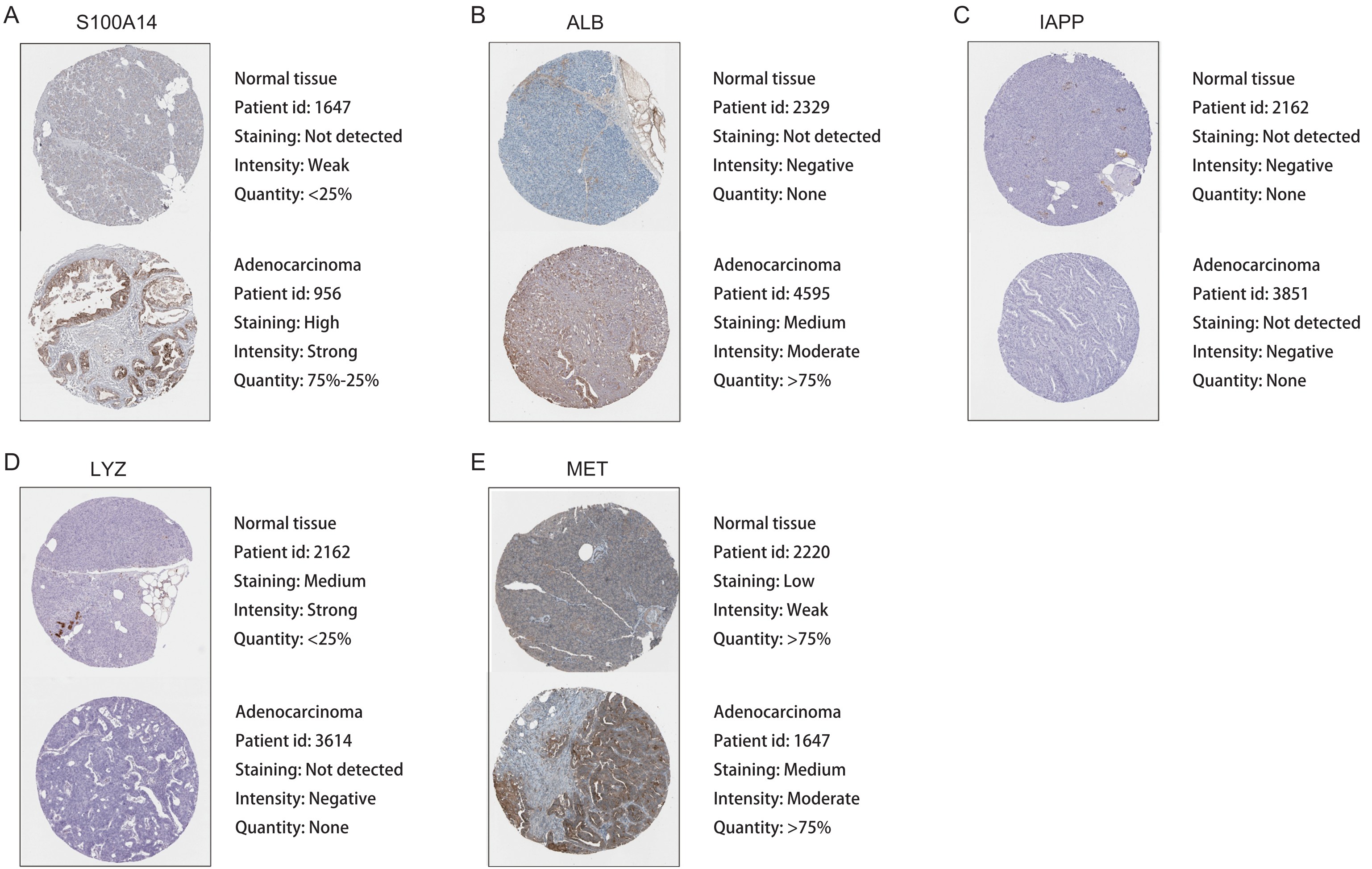

Figure S3. Representative images of hub IRGs expression status except CXCL10 and LIFR in normal pancreas and PC tissues. (The expression information of CXCL10 and LIFR was unavailable in HPA database)

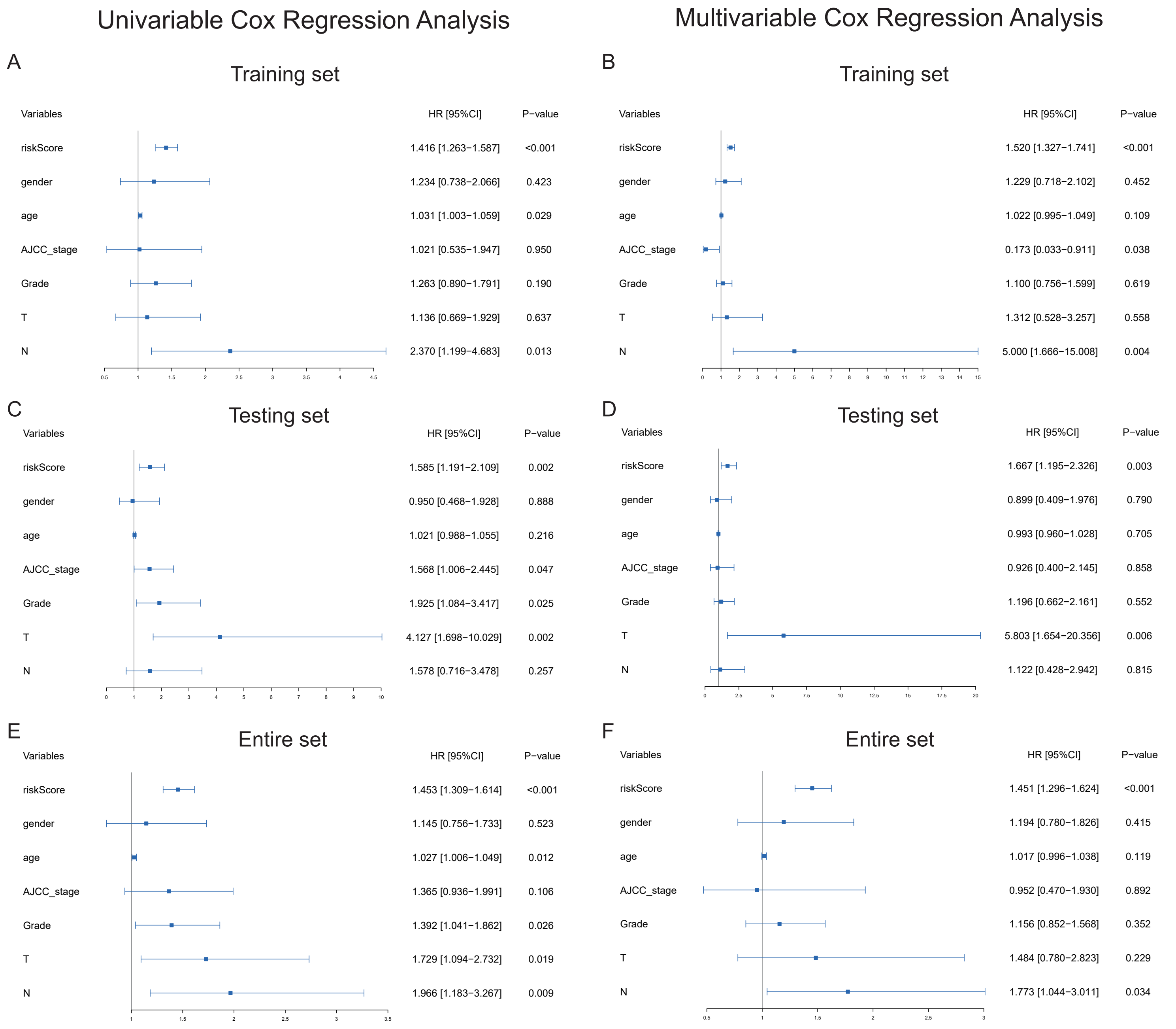

Figure S4. Independence of the risk signature and the other clinical variables, including, age, tumor grade, AJCC_stage and gender. (A, C, E) Univariate Cox regression analyses in the training set, testing set and entire set. (B, D, F) Multivariate Cox regression analyses in the training set testing set and entire set.

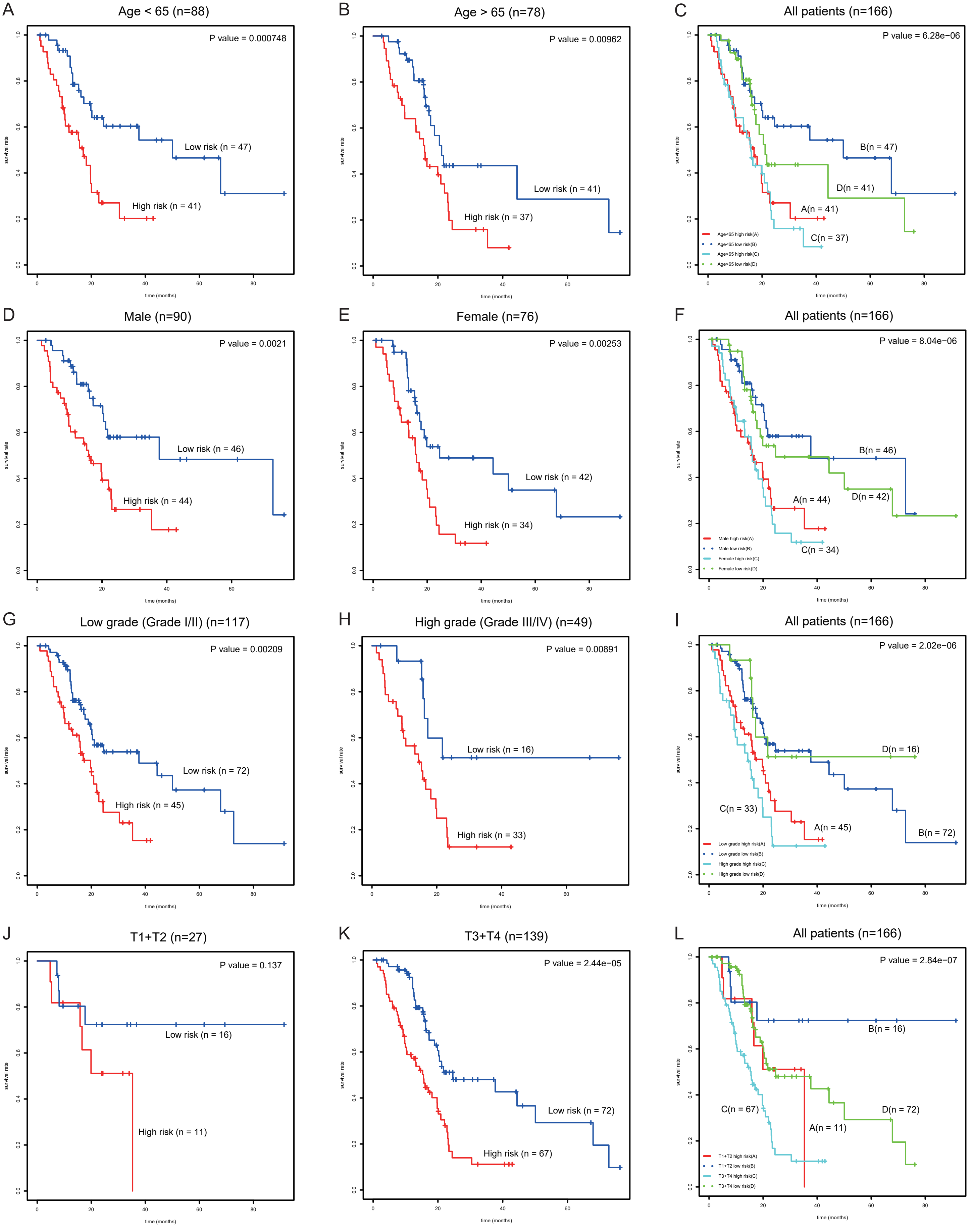

Figure S5. Stratification analyses of all patients using the risk signature. (A-C) The Kaplan-Meier analysis of the younger stratum (age ≤ 65, n=88), older stratum (age >65, n=78) and all patients with PC (n=166). (D-F) The Kaplan-Meier analysis of the male stratum (n=90), female stratum n=76) and all patients with PC (n=166). (G-I) The Kaplan-Meier analysis of the Grade Ⅰ/Ⅱ stratum (n=117), Grade Ⅲ/Ⅳ stratum (n=49) and all patients with PC (n=166). (J-L) The Kaplan-Meier analysis of the T1+T2 stratum (n=27), T3+T4 stratum (n=139) and all patients with PC (n=166).

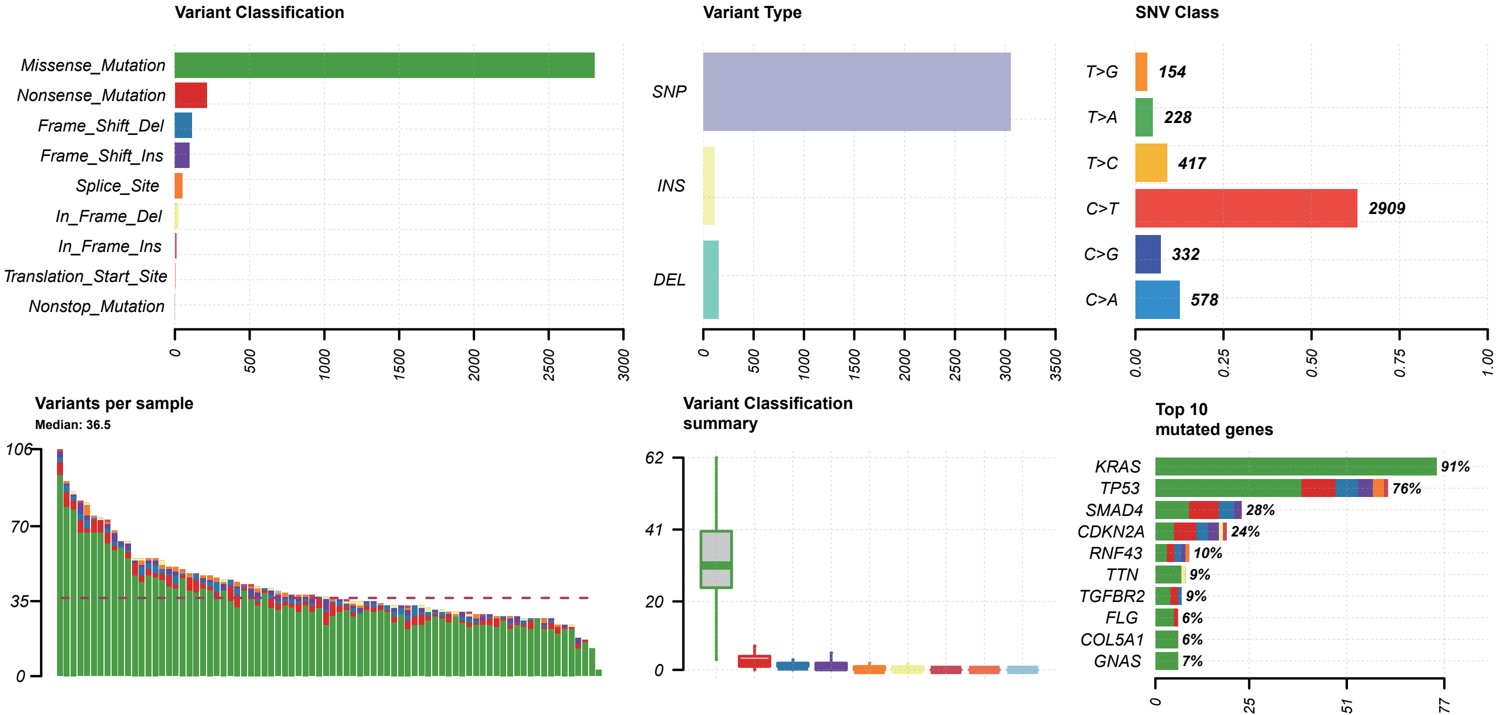

Figure S6: The mutation profiles of patients in S100A14 high expression group.

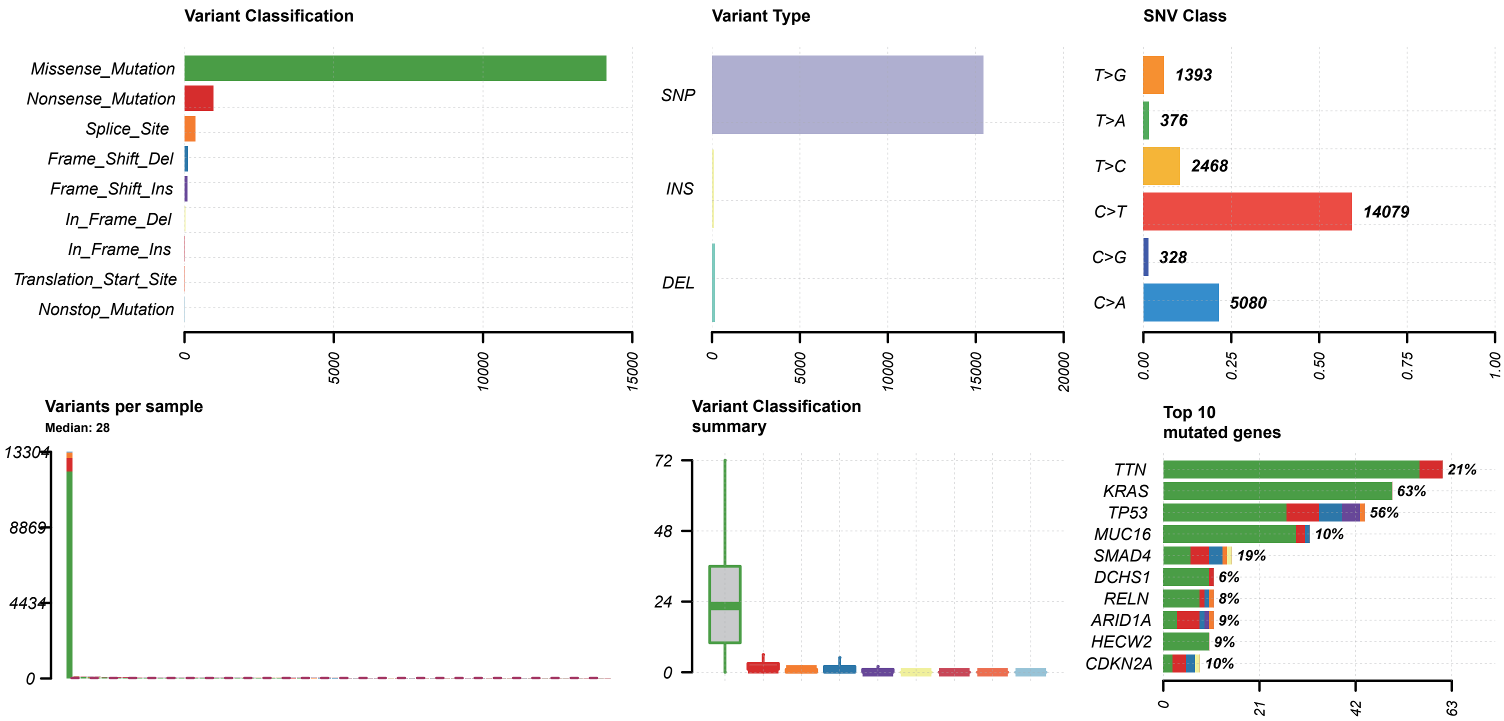

Figure S7: The mutation profiles of patients in S100A14 low expression group.

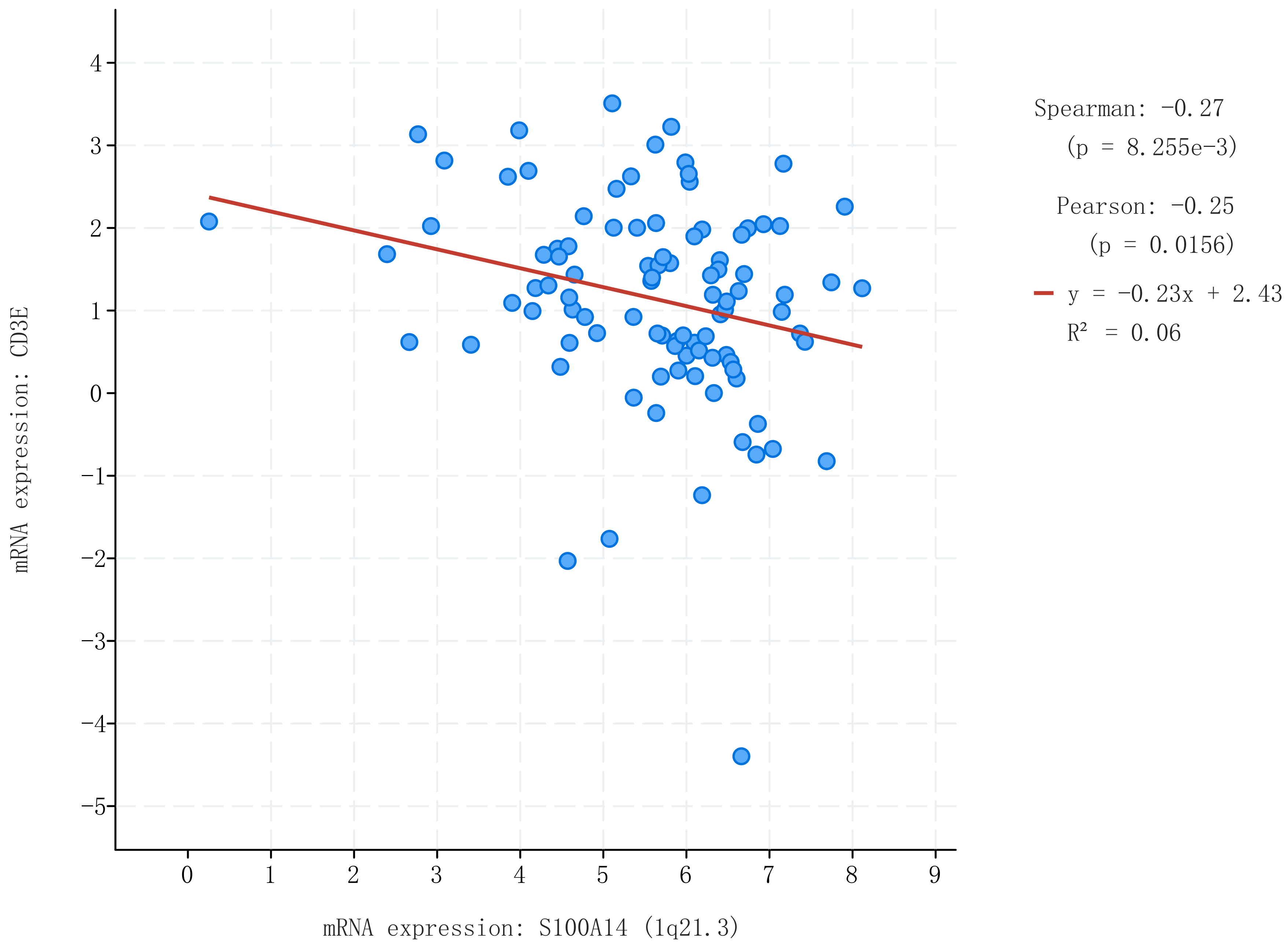

Figure S8: Correlation analysis of S100A14 and CD3E in the Bailey P. et al. cohort.
