# Supplementary figures and images for: Construction of immune-related signature and identification of S100A14 determining immune-suppressive microenvironment in pancreatic cancer
Source: BMC Cancer. 2022 Aug 11;22:879. doi: 10.1186/s12885-022-09927-0 (PMC9367131; doi:10.1186/s12885-022-09927-0)

Replicate 1

S100A14

$\beta$ -actin

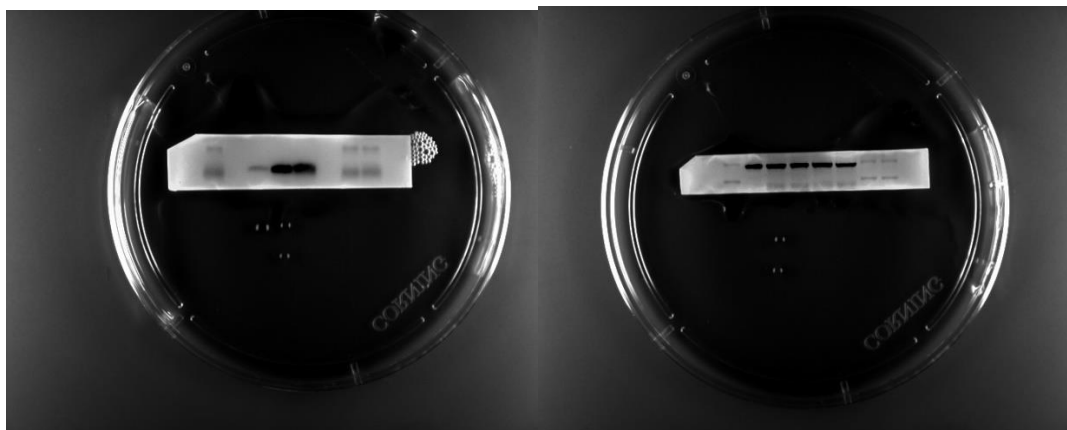

Replicate 2

S100A14

$\beta$ -actin

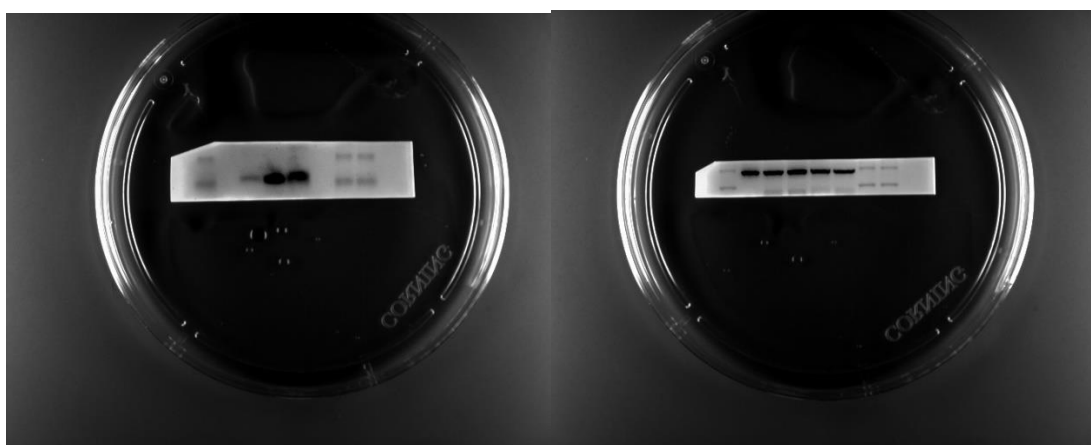

Replicate 3

S100A14

$\beta$ -actin

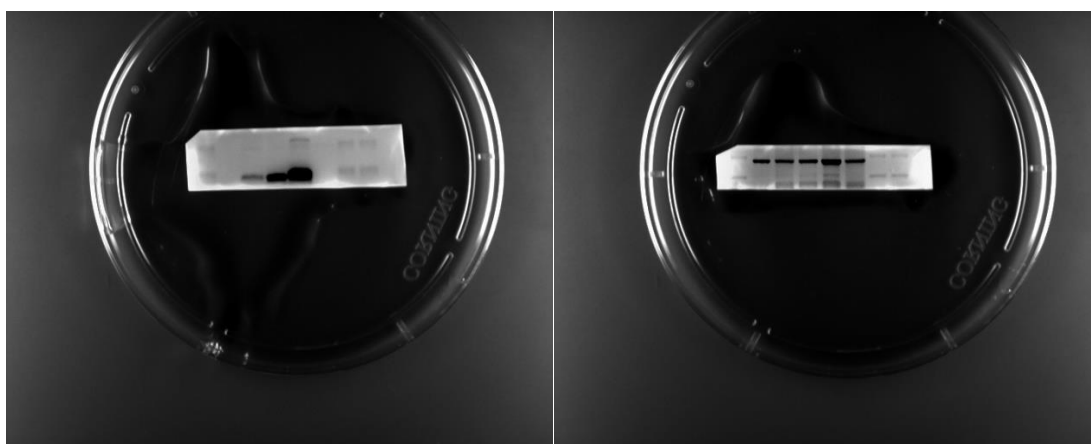

Supplement: Supplementary file 2 — Additional file 2. [file 12885_2022_9927_MOESM2_ESM.pdf]
