# Supplementary material for: Construction of immune-related signature and identification of S100A14 determining immune-suppressive microenvironment in pancreatic cancer
Source: BMC Cancer. 2022 Aug 11;22:879. doi: 10.1186/s12885-022-09927-0 (PMC9367131; doi:10.1186/s12885-022-09927-0)
Supplement: Supplementary file 3 — Additional file 3: Table S1. Detailed clinical and pathologic information of TCGA entire set. Table S2. Detailed clinical and pathologic information of CRA001160 dataset. Table S3. The imaging channels for each antibody and antibody incubation information in tissue microenvironment landscape imaging. Table S4. Immune-related genes obtained from the ImmPort database. [file 12885_2022_9927_MOESM3_ESM.pdf]

Table S1. Detailed clinical and pathologic information of TCGA entire set.

status	life_span	gender	age	AJCC_stage	Grade	T	N	M	
TCGA-2J-AAB1	1	66	1	65	2	3	T3	N1	M0
TCGA-2J-AAB4	0	729	1	48	2	2	T2	N1	M0
TCGA-2J-AAB6	1	293	1	75	2	2	T3	N0	M0
TCGA-2J-AAB8	0	80	1	71	2	3	T3	N1	M0
TCGA-2J-AAB9	1	627	2	70	2	1	T3	N1	M0
TCGA-2J-AABA	1	607	1	55	2	2	T3	N1	M0
TCGA-2J-AABE	0	676	1	73	2	2	T3	N0	M0
TCGA-2J-AABF	1	691	1	73	2	3	T3	N1	M0
TCGA-2J-AABH	0	1287	1	61	2	3	T3	N0	M0
TCGA-2J-AABI	0	969	2	55	2	3	T3	N0	M0
TCGA-2J-AABK	0	484	1	71	2	2	T3	N1	M0
TCGA-2J-AABO	0	440	1	43	2	2	T3	N1	M0
TCGA-2J-AABP	0	463	2	58	2	4	T3	N1	M0
TCGA-2J-AABR	0	438	2	60	2	3	T3	N0	M0
TCGA-2J-AABT	0	319	2	72	2	2	T3	N1	M0
TCGA-2J-AABU	1	277	1	56	2	3	T3	N1	M0
TCGA-2J-AABV	1	652	1	74	2	4	T3	N1	M0
TCGA-2L-AAQA	1	143	1	76	2	2	T3	N1	MX
TCGA-2L-AAQE	1	684	1	56	2	2	T3	N1	M0
TCGA-2L-AAQI	1	103	1	66	2	3	T3	N1	MX
TCGA-2L-AAQJ	1	394	2	49	3	2	T4	N0	MX
TCGA-2L-AAQL	1	292	1	82	2	3	T3	N1	MX
TCGA-2L-AAQM	0	1383	1	52	2	1	T3	N1	MX
TCGA-3A-A9I7	0	1323	1	45	2	2	T3	N1	MX
TCGA-3A-A9I9	1	634	1	67	2	2	T3	N0	MX
TCGA-3A-A9IB	1	224	2	69	2	3	T3	N1	MX
TCGA-3A-A9IC	1	738	2	61	2	2	T3	N1	MX
TCGA-3A-A9IH	0	1021	2	66	1	2	T1	N0	MX
TCGA-3A-A9IJ	0	1854	1	65	1	1	T2	N0	MX
TCGA-3A-A9IL	0	2741	2	39	1	1	T1	N1	MX
TCGA-3A-A9IN	0	2084	2	62	1	2	T2	NX	MX
TCGA-3A-A9IR	0	1542	2	64	1	1	T2	N0	MX
TCGA-3A-A9IS	0	998	1	67	1	1	T2	N0	MX
TCGA-3A-A9IU	1	458	1	65	2	3	T3	N1	MX
TCGA-3A-A9IV	0	1103	2	59	1	1	T2	N0	MX
TCGA-3A-A9IX	0	1037	1	40	1	2	T1	N0	MX
TCGA-3A-A9IZ	1	308	1	47	2	2	T3	N1	MX
TCGA-3A-A9J0	0	743	1	75	2	2	T3	N1	MX
TCGA-3E-AAAY	0	2285	1	67	2	3	T3	N1	MX
TCGA-3E-AAAZ	1	2182	1	71	2	2	T3	N0	MX
TCGA-F2-6879	1	334	1	57	2	2	T3	N1	M0
TCGA-F2-6880	0	295	1	70	2	1	T3	N1	M0
TCGA-F2-7273	1	592	1	54	2	3	T3	N1	M0
TCGA-F2-7276	1	216	1	64	2	1	T3	N1	M0
TCGA-F2-A44G	1	233	2	79	2	2	T3	N1	M0
TCGA-F2-A44H	0	586	1	65	2	2	T3	N0	M0
TCGA-F2-A7TX	1	95	1	77	2	3	T3	N1	M0
TCGA-F2-A8YN	0	517	1	76	2	2	T3	N0	M0
TCGA-FB-A4P5	1	179	2	69	2	2	T3	N1	MX
TCGA-FB-A4P6	0	767	1	54	2	1	T3	N1	MX
TCGA-FB-A545	1	732	2	72	2	2	T3	N1	M0
TCGA-FB-A5VM	1	498	1	74	1	3	T2	N0	M0
TCGA-FB-A78T	1	375	2	71	2	2	T3	N1	M0
TCGA-FB-A7DR	1	353	1	48	2	2	T3	NX	M0
TCGA-FB-AAPP	1	485	1	71	2	3	T3	N1	MX
TCGA-FB-AAPQ	1	1130	1	65	2	2	T3	N1	MX
TCGA-FB-AAPS	0	228	2	62	2	2	T2	N1	MX
TCGA-FB-AAPU	1	381	2	41	2	2	T3	N0	MX

TCGA-FB-AAPY	1	1059	1	71	2	2	T2	N1	MX
TCGA-FB-AAPZ	0	716	1	54	2	3	T2	N1	MX
TCGA-FB-AAQ0	1	473	1	68	2	3	T3	N0	MX
TCGA-FB-AAQ1	1	123	1	49	2	2	T3	N1	MX
TCGA-FB-AAQ2	1	153	2	81	2	3	T3	N1	MX
TCGA-FB-AAQ3	1	31	2	65	2	2	T3	N1	MX
TCGA-FB-AAQ6	1	244	1	85	1	2	T1	N0	MX
TCGA-H6-8124	0	392	2	56	2	3	T3	N1	M0
TCGA-H6-A45N	1	397	2	88	2	3	T3	N1	MX
TCGA-H8-A6C1	0	671	1	53	2	2	T3	N0	MX
TCGA-HV-A5A3	1	128	1	50	2	2	T3	NX	MX
TCGA-HV-A5A4	0	232	2	72	2	2	T3	N0	MX
TCGA-HV-A5A5	0	289	2	61	2	2	T3	N1	M0
TCGA-HV-A5A6	1	2036	2	65	2	1	T3	N1	M0
TCGA-HV-A7OL	0	252	1	70	2	2	T3	N0	MX
TCGA-HV-AA8V	0	920	1	50	2	3	T3	N1	MX
TCGA-HV-AA8X	1	532	2	75	2	2	T2	N1	MX
TCGA-HZ-7289	1	661	1	77	2	1	T3	N1	MX
TCGA-HZ-7918	0	969	1	72	2	3	T3	N1	M0
TCGA-HZ-7919	1	593	2	52	2	2	T3	N1	M0
TCGA-HZ-7920	1	236	1	71	1	2	T2	N0	MX
TCGA-HZ-7923	0	314	1	65	2	2	T3	N0	MX
TCGA-HZ-7924	0	840	2	60	2	2	T3	N0	MX
TCGA-HZ-7925	1	614	1	66	2	2	T3	N1	MX
TCGA-HZ-7926	1	518	1	57	2	1	T3	N1	MX
TCGA-HZ-8001	0	706	1	69	3	2	T4	N0	MX
TCGA-HZ-8002	1	366	1	61	2	2	T3	N1	MX
TCGA-HZ-8003	1	596	2	65	2	2	T3	N1	MX
TCGA-HZ-8005	1	120	1	81	2	3	T3	N1	MX
TCGA-HZ-8315	1	299	2	54	2	2	T3	N0	MX
TCGA-HZ-8317	1	378	2	69	2	1	T3	N1	MX
TCGA-HZ-8519	0	454	1	73	1	3	T1	N0	MX
TCGA-HZ-8636	1	545	2	58	4	3	T3	N0	M1
TCGA-HZ-8637	1	517	2	76	2	3	T3	N1	MX
TCGA-HZ-A49G	0	660	2	58	2	2	T2	N1	MX
TCGA-HZ-A49H	0	491	2	68	2	2	T3	N1	MX
TCGA-HZ-A49I	1	308	1	77	2	2	T3	N1	MX
TCGA-HZ-A4BH	0	194	1	75	2	3	T3	N1	MX
TCGA-HZ-A4BK	0	657	1	72	2	3	T3	N1	MX
TCGA-HZ-A77O	1	160	2	77	2	2	T2	N1	MX
TCGA-HZ-A77P	0	330	1	77	2	1	T3	N1	MX
TCGA-HZ-A77Q	0	33	2	55	2	2	T3	N1	MX
TCGA-HZ-A9TJ	0	603	1	70	4	2	T3	N0	M1
TCGA-IB-7644	1	394	2	65	4	2	T3	N1	M1
TCGA-IB-7645	1	1502	2	44	2	1	T3	N1	M0
TCGA-IB-7646	1	145	1	60	2	2	T3	N1	M0
TCGA-IB-7649	1	467	2	73	2	2	T3	N1	M0
TCGA-IB-7651	1	603	2	64	2	2	T3	N1	M0
TCGA-IB-7652	0	1116	2	49	2	2	T3	N1	M0
TCGA-IB-7654	1	476	1	80	2	2	T2	N1	M0
TCGA-IB-7885	0	1257	2	78	2	2	T3	N1	M0
TCGA-IB-7886	1	123	1	80	2	3	T3	N1	M0
TCGA-IB-7887	1	110	2	62	2	2	T3	N1	M0
TCGA-IB-7888	1	1332	2	66	2	2	T3	N0	M0
TCGA-IB-7889	1	481	2	85	2	1	T3	N1	M0
TCGA-IB-7890	1	598	1	73	1	3	T2	N0	M0
TCGA-IB-7891	1	913	2	49	2	1	T3	N1	M0
TCGA-IB-7893	1	117	1	64	2	3	T3	N0	M0
TCGA-IB-7897	1	486	2	53	2	2	T3	N1	M0
TCGA-IB-8126	0	462	2	79	3	1	T4	N1	M0
TCGA-IB-8127	0	522	1	59	2	2	T3	N1	M0

TCGA-IB-A5SO	1	365	1	71	2	2	T3	N1	M0
TCGA-IB-A5SP	0	482	1	77	2	2	T3	N0	M0
TCGA-IB-A5SQ	1	219	2	56	1	2	T2	N0	M0
TCGA-IB-A5SS	1	460	2	64	2	3	T3	N1	M0
TCGA-IB-A5ST	0	635	2	64	2	2	T3	N1	M0
TCGA-IB-A6UF	0	666	1	63	2	2	T3	N1	M0
TCGA-IB-A6UG	1	41	1	65	2	3	T3	N1	M0
TCGA-IB-A7LX	1	250	1	57	2	2	T3	N1	MX
TCGA-IB-A7M4	0	483	1	81	2	3	T3	N1	MX
TCGA-IB-AAUN	1	144	2	74	1	2	T2	N0	M0
TCGA-IB-AAUO	1	239	2	64	2	3	T3	N1	M0
TCGA-IB-AAUP	0	431	1	68	2	2	T3	N1	M0
TCGA-IB-AAUQ	1	183	1	50	2	2	T3	N1	M0
TCGA-IB-AAUR	0	338	1	67	2	1	T3	N1	M0
TCGA-IB-AAUS	0	225	2	84	2	2	T3	N1	M0
TCGA-IB-AAUT	0	287	1	65	2	1	T2	N1	M0
TCGA-IB-AAUU	0	245	1	35	2	3	T3	N1	M0
TCGA-IB-AAUV	0	404	1	49	2	2	T3	N1	M0
TCGA-IB-AAUW	1	230	2	63	2	3	T3	N1	M0
TCGA-L1-A7W4	1	278	2	48	2	3	T3	N1	M0
TCGA-LB-A7SX	1	393	2	74	2	2	T3	N1	MX
TCGA-LB-A8F3	0	379	2	64	2	1	T3	N0	MX
TCGA-LB-A9Q5	1	313	2	63	2	3	T3	N1	MX
TCGA-M8-A5N4	0	584	2	48	2	2	T3	N0	M0
TCGA-OE-A75W	1	267	1	75	2	1	T3	N0	M0
TCGA-PZ-A5RE	1	470	2	44	2	3	T3	N1	MX
TCGA-Q3-A5QY	0	416	1	58	2	2	T3	N1	MX
TCGA-Q3-AA2A	0	95	2	64	1	1	T3	N1	MX
TCGA-RB-A7B8	1	466	2	81	2	2	T3	N1	MX
TCGA-RB-AA9M	0	286	1	43	2	3	T1	N1	MX
TCGA-S4-A8RM	0	737	1	67	2	3	T3	N1	MX
TCGA-S4-A8RO	0	525	2	75	2	2	T3	N1	MX
TCGA-S4-A8RP	1	702	2	77	2	3	T3	N1	MX
TCGA-US-A774	1	695	2	76	2	3	T3	N1	MX
TCGA-US-A776	0	1216	1	61	2	2	T3	N0	MX
TCGA-US-A779	1	511	2	54	2	1	T3	N1	MX
TCGA-US-A77E	1	430	1	73	2	3	T3	N1	MX
TCGA-US-A77J	1	568	2	81	2	2	T3	N1	MX
TCGA-XD-AAUG	0	420	2	66	4	2	T3	N1	M1
TCGA-XD-AAUH	0	395	2	57	2	2	T3	N1	M0
TCGA-XD-AAUI	1	366	2	50	2	2	T3	N1	MX
TCGA-XD-AAUL	0	498	1	56	2	2	T3	N0	MX
TCGA-XN-A8T3	0	951	1	67	1	2	T2	N0	M0
TCGA-XN-A8T5	0	720	2	53	1	2	T2	N0	M0
TCGA-YB-A89D	0	350	1	59	2	2	T3	N1	MX
TCGA-YY-A8LH	0	2016	2	61	2	3	T3	N1	MX
TCGA-Z5-AAPL	0	467	2	74	2	1	T3	N0	M0

Table S2. Detailed clinical and pathologic information of CRA001160 dataset.

Number	Gender	Age(y/o)	Diabetes Mellitus	TNM Classification	Staging
PUMCH-T1	M	64	N	T4N2M0	III
PUMCH-T2	M	52	N	T1cN1M0	IIB
PUMCH-T3	F	58	Y	T2N0M0	IB
PUMCH-T4	F	72	Y	T1cN1M0	IIB
PUMCH-T5	F	65	Y	T2N0M0	IB
PUMCH-T6	M	64	N	T3N0M0	IIA
PUMCH-T7	M	70	Y	T3N1M0	IIB
PUMCH-T8	F	66	N	T1cN2M0	III
PUMCH-T9	M	36	N	T2N0M0	IIA
PUMCH-T10	M	61	Y	T2N1M0	IB
PUMCH-T11	M	51	N	T3N1M0	IIB
PUMCH-T12	M	54	N	T3N2M0	III
PUMCH-T13	F	58	Y	T2N1M0	IIB
PUMCH-T14	F	67	Y	T2N1M0	IIB
PUMCH-T15	F	54	N	T2N1M0	IIB
PUMCH-T16	F	56	N	T2N1M0	IIB
PUMCH-T17	F	71	N	T2N0M0	IB
PUMCH-T18	F	68	Y	T2N0M0	IB
PUMCH-T19	F	59	N	T2N0M0	IB
PUMCH-T20	M	59	N	T3N1M0	IIB
PUMCH-T21	M	59	Y	T2N0M0	IB
PUMCH-T22	F	67	N	T2N0M0	IB
PUMCH-T23	M	54	Y	T2N1M0	IIB
PUMCH-T24	F	44	N	T1cN0M0	IB

Table S3. The imaging channels for each antibody and antibody incubation information in tissue microenvironment landscape imaging.

Primary antibody	Catalogue number	Provider	Concentration	Incubation time	Channel
anti-CD3	60181-1-Ig	proteintech	1:10000	1h	opal 540
anti-S100A14	10489-1-AP	proteintech	1:10000	1h	opal 620
anti-CD8	66868-1-Ig	proteintech	1:20000	1h	opal 690

Table S4. Immune-related genes obtained from the ImmPort database.

	Symbol
1	AZGP1
2	B2M
3	CALR
4	CANX
5	CD1A
6	CD1B
7	CD1C
8	CD1D
9	CD1E
10	CD4
11	CD8A
12	CD8B
13	CD74
14	CREB1
15	CTSB
16	CTSE
17	CTSL
18	CTSS
19	FCER1G
20	FCGRT
21	PDIA3
22	HFE
23	HLA-A
24	HLA-B
25	HLA-C
26	HLA-DMA
27	HLA-DMB
28	HLA-DOA
29	HLA-DOB
30	HLA-DPA1
31	HLA-DPB1
32	HLA-DQA1
33	HLA-DQA2
34	HLA-DQB1
35	HLA-DRA
36	HLA-DRB1
37	HLA-DRB3
38	HLA-DRB4
39	HLA-DRB5
40	HLA-E
41	HLA-F
42	HLA-G
43	HLA-H
44	MR1
45	HSPA1A
46	HSPA1B
47	HSPA1L
48	HSPA2
49	HSPA4
50	HSPA5
51	HSPA6
52	HSPA8
53	HSP90AA1
54	HSP90AB1
55	ICAM1

56	IFNA1
57	IFNA2
58	IFNA4
59	IFNA5
60	IFNA6
61	IFNA7
62	IFNA8
63	IFNA10
64	IFNA13
65	IFNA14
66	IFNA16
67	IFNA17
68	IFNA21
69	IFNG
70	KIR2DL1
71	KIR2DL2
72	KIR2DL3
73	KIR2DL4
74	KIR2DS1
75	KIR2DS3
76	KIR2DS4
77	KIR2DS5
78	KIR3DL1
79	KIR3DL2
80	KLRC1
81	KLRC2
82	KLRC3
83	KLRD1
84	LTA
85	CIITA
86	MICA
87	MICB
88	NFYA
89	NFYB
90	NFYC
91	LGMN
92	PSMB8
93	PSMC1
94	PSMC2
95	PSMC3
96	PSMC4
97	PSMC5
98	PSMC6
99	PSMD1
100	PSMD2
101	PSMD3
102	PSMD4
103	PSMD5
104	PSMD7
105	PSMD8
106	PSMD10
107	PSMD11
108	PSMD13
109	PSME1
110	PSME2
111	RELB
112	RFX5

113	RFXAP
114	SLC10A2
115	TAP1
116	TAP2
117	TAPBP
118	THBS1
119	SEM1
120	KLRC4
121	AP3B1
122	RFXANK
123	PSMD6
124	PSME3
125	PSMD14
126	CLEC4M
127	IFI30
128	PROCR
129	ADRM1
130	ECPAS
131	TRPC4AP
132	CD209
133	UBXN1
134	ERAP1
135	TAPBPL
136	KIR2DL5A
137	ERAP2
138	ULBP3
139	ULBP2
140	ULBP1
141	KIR3DL3
142	RAET1E
143	RAET1L
144	UBR1
145	RAET1G
146	PDIA2
147	HAMP
148	PI3
149	CAMP
150	DEFB4A
151	PPBP
152	REG3G
153	CXCL14
154	CXCL16
155	SLPI
156	CXCL8
157	CXCL10
158	CXCL9
159	CXCL5
160	CXCL11
161	CXCL6
162	CXCL1
163	CXCL12
164	CXCL13
165	CXCL2
166	PF4
167	XCL1
168	CXCL3
169	DEFB103B

170 CCL13
171 CCL1
172 DEFB1
173 CCL8
174 ELANE
175 DEFB103A
176 DEFA3
177 DEFA1
178 TMSB10
179 DEFA6
180 DEFA5
181 DEFA4
182 LCN2
183 LCN1
184 COLEC10
185 BPI
186 S100A9
187 S100A8
188 DCD
189 LCN6
190 S100A12
191 HTN3
192 LCN8
193 DEFA1B
194 CCR10
195 CELA1
196 DEFB106A
197 PENK
198 BPIFC
199 MMP12
200 BPIFB6
201 LEAP2
202 SFTPD
203 LCN9
204 BPIFB2
205 PTGDS
206 TMSB4X
207 PGLYRP1
208 ZC3HAV1
209 TMSB15A
210 S100B
211 S100A13
212 S100A6
213 DEFB119
214 DEFB107A
215 DEFB105A
216 SERPIND1
217 DEFB129
218 DEFB127
219 S100P
220 S100A7
221 DEFB104A
222 DEFB126
223 DEFB106B
224 DEFB104B
225 DEFB107B
226 PGLYRP3

227	PGLYRP2
228	S100A10
229	S100A2
230	DEFB125
231	DEFB123
232	DEFB105B
233	DEFB132
234	BPIFB3
235	LCN12
236	PGLYRP4
237	S100A11
238	S100A5
239	S100A3
240	S100A1
241	DEFB128
242	DEFB108B
243	HTN1
244	LMBR1L
245	S100A7A
246	DEFB118
247	COLEC12
248	TMSB4Y
249	DEFB131A
250	DEFB134
251	DEFB130A
252	DEFB124
253	DEFB121
254	DEFB116
255	DEFB115
256	DEFB114
257	DEFB113
258	DEFB112
259	DEFB110
260	TMSB15B
261	DEFB133
262	S100Z
263	MAVS
264	TMSB4XP8
265	S100A14
266	LCN10
267	S100A16
268	DEFB136
269	DEFB135
270	DEFB117
271	ZC3HAV1L
272	S100A7L2
273	MBL3P
274	DEFB4B
275	BPIFB4
276	IFNAR1
277	AZU1
278	DEFB131B
279	DEFA1A3
280	LCN1P1
281	S100G
282	DEFA7P
283	DEFB130B

284	DEFB108F
285	DEFB131C
286	TCHHL1
287	TINAGL1
288	IFNGR1
289	SLC22A17
290	WFIKKN1
291	WFDC2
292	IL6
293	UMODL1
294	TGFB1
295	PF4V1
296	MMP9
297	ANOS1
298	TLR4
299	SPAG11B
300	A2M
301	NFKB1
302	APOBEC3G
303	FABP6
304	NOD2
305	MBL2
306	SFTPA1
307	RBP1
308	TLR2
309	SLC40A1
310	PLAU
311	IL1B
312	PAEP
313	HJV
314	MUC5AC
315	OBP2A
316	PLTP
317	MX1
318	DDX58
319	IFNL1
320	IRF3
321	SFTPA2
322	LPA
323	LBP
324	RBP4
325	NOX4
326	LTF
327	IFNB1
328	RBP5
329	FABP7
330	FABP5
331	FABP3
332	FABP2
333	FABP4
334	R3HDML
335	BPIFA3
336	BPIFB1
337	OASL
338	CRABP2
339	CRABP1
340	RBP7

341	DUOX1
342	OBP2B
343	RBP2
344	LCN15
345	CETP
346	FABP12
347	FABP9
348	BPIFA1
349	LCNL1
350	C8G
351	SPAG11A
352	PI15
353	NOX1
354	PMP2
355	APOD
356	ORM2
357	ORM1
358	TNF
359	CTSG
360	PRTN3
361	MAPK1
362	PML
363	AEN
364	CYBB
365	BPIFA2
366	ISG20
367	BCL3
368	ISG20L2
369	NOX5
370	NOX3
371	DUOX2
372	TLR3
373	TFRC
374	IFIH1
375	LRP1
376	TRIM5
377	IDO1
378	GDF15
379	NEDD4
380	ADIPOQ
381	STAT3
382	STAT1
383	IFNL2
384	SOCS3
385	SEMG1
386	TNFSF10
387	CCL20
388	SOCS1
389	RNASEL
390	IRF1
391	IL15
392	APOBEC3F
393	PLAAT4
394	CHIT1
395	CD40
396	TLR7
397	PPIA

398 ZYX
399 NLRX1
400 PGC
401 VEGFA
402 IKBKE
403 ISG15
404 DHX58
405 TNFAIP3
406 TFR2
407 FCN2
408 MUC4
409 F2R
410 ELN
411 IL27
412 MAPT
413 LYZ
414 CCL5
415 LEP
416 CYLD
417 KLKB1
418 CST4
419 CSRP1
420 MAPK14
421 JUN
422 ITGAV
423 IRF5
424 CCR6
425 IL12B
426 TLR8
427 GNLY
428 CD81
429 EIF2AK2
430 APOM
431 CACYBP
432 NOD1
433 MAPK8
434 MAPK3
435 BST2
436 BPHL
437 PLA2G2A
438 GRN
439 NEWENTRY
440 PDGFRA
441 GNAI1
442 WNT5A
443 FURIN
444 ADAR
445 TYK2
446 NOS2
447 TRAF3
448 TPT1
449 TPM2
450 NEO1
451 AHNAK
452 TLR1
453 TK2
454 PRDX2

455	MX2
456	FGF2
457	FGA
458	TCF7L2
459	F2RL1
460	TKFC
461	MSR1
462	NFKBIZ
463	LMBR1
464	EPPIN
465	SRC
466	MPO
467	ELAVL1
468	ROBO3
469	SP1
470	SOD1
471	PDF
472	DLL4
473	ECD
474	SLC11A1
475	DMBT1
476	STING1
477	SKIV2L
478	SEMG2
479	DES
480	DCK
481	DAXX
482	TNFRSF10A
483	TNFRSF10B
484	EED
485	CCL4
486	LIMS1
487	LALBA
488	APOBEC3H
489	TMPRSS6
490	SPINK5
491	MARCO
492	BECN1
493	TNFSF11
494	KNG1
495	CSK
496	KLRK1
497	KCNH2
498	JUND
499	JAK1
500	CLDN4
501	CCL28
502	RNASE3
503	RN7SL1
504	IRF7
505	IREB2
506	ILK
507	IL18
508	IL17A
509	LTB4R
510	APOBEC3A
511	MASP2

512	TRIM27
513	RELA
514	IL7R
515	IL1A
516	PTX3
517	IFNAR2
518	IFN1@
519	SYTL1
520	APOBEC3C
521	DDX17
522	PTGS2
523	HTR1A
524	SEPTIN7
525	CD40LG
526	CD14
527	MASP1
528	PROC
529	MAP2K2
530	MAP2K1
531	HRG
532	NDRG1
533	IRF9
534	TRIM22
535	LANCL1
536	PPP4C
537	HMOX1
538	HMGB1
539	RNASE7
540	ABCC4
541	HGF
542	HDAC1
543	IFNLR1
544	PLSCR1
545	BACH2
546	TANK
547	PIK3CG
548	ARRB1
549	RSAD2
550	STAB2
551	TBK1
552	PDYN
553	PDGFRB
554	PDCD1
555	PCSK2
556	PCSK1
557	ARG2
558	AQP9
559	FASLG
560	APOH
561	BIRC5
562	ANXA6
563	IL22
564	VTN
565	VIM
566	VCAM1
567	PRDX1
568	GFAP

569	GBP2
570	ALB
571	SLC29A3
572	OAS1
573	AGER
574	UNC93B1
575	TNFSF4
576	NOS1
577	ACTG1
578	ACTA1
579	ACO1
580	SERPINA3
581	CXCR1
582	CCL15
583	CCL14
584	CCL16
585	CCL19
586	CCL18
587	CCL17
588	CCL26
589	CCL22
590	CCR3
591	CCL4L1
592	ACKR2
593	CCR7
594	CCL27
595	CCR8
596	ACKR4
597	CCL2
598	CCL21
599	CCL7
600	CCL3
601	CCL11
602	CCR5
603	CCL23
604	CCL25
605	CCL3L3
606	CCL4L2
607	CCL3L1
608	CCR1
609	CCL24
610	XCL2
611	CXCR4
612	CXCR6
613	CCR4
614	TAFA5
615	TAFA3
616	TAFA4
617	TAFA1
618	TAFA2
619	CCL15-CCL14
620	PTK2B
621	IL4
622	CDH1
623	LTBP1
624	IL13
625	IL10

626	IL2
627	PPARG
628	FGR
629	MIF
630	CRP
631	JAK2
632	PTK2
633	PTGDR
634	CD86
635	HCK
636	VDR
637	OLR1
638	GRK2
639	TXK
640	RNASE2
641	CD79A
642	CD79B
643	LYN
644	SYK
645	BTK
646	BLNK
647	VAV3
648	VAV1
649	VAV2
650	RAC1
651	RAC2
652	RAC3
653	PPP3CA
654	PPP3CB
655	PPP3CC
656	CHP1
657	PPP3R1
658	PPP3R2
659	CHP2
660	NFAT5
661	NFATC1
662	NFATC2
663	NFATC3
664	NFATC4
665	HRAS
666	KRAS
667	NRAS
668	FOS
669	CARD11
670	BCL10
671	MALT1
672	CHUK
673	IKBKB
674	IKBKG
675	NFKBIA
676	NFKBIB
677	NFKBIE
678	CD19
679	CR2
680	PIK3R5
681	PIK3R1
682	PIK3R2

683	PIK3R3
684	PIK3CA
685	PIK3CB
686	PIK3CD
687	AKT3
688	AKT1
689	AKT2
690	GSK3B
691	INPP5D
692	CD22
693	CD72
694	PTPN6
695	LILRB3
696	FCGR2B
697	RASGRP3
698	PLCG2
699	PRKCB
700	IFITM1
701	IGH
702	IGHA1
703	IGHA2
704	IGHD
705	IGHD1-1
706	IGHD1-14
707	IGHD1-20
708	IGHD1-26
709	IGHD1-7
710	IGHD2-15
711	IGHD2-2
712	IGHD2-21
713	IGHD2-8
714	IGHD3-10
715	IGHD3-16
716	IGHD3-22
717	IGHD3-3
718	IGHD3-9
719	IGHD4-11
720	IGHD4-17
721	IGHD4-23
722	IGHD4-4
723	IGHD5-12
724	IGHD5-18
725	IGHD5-24
726	IGHD5-5
727	IGHD6-13
728	IGHD6-19
729	IGHD6-25
730	IGHD6-6
731	IGHD7-27
732	IGHE
733	IGHG1
734	IGHG2
735	IGHG3
736	IGHG4
737	IGHJ1
738	IGHJ2
739	IGHJ3

740	IGHJ4
741	IGHJ5
742	IGHJ6
743	IGHM
744	IGHV1-18
745	IGHV1-2
746	IGHV1-24
747	IGHV1-3
748	IGHV1-45
749	IGHV1-46
750	IGHV1-58
751	IGHV1-69
752	IGHV1-8
753	IGHV1-38-4
754	IGHV1-69-2
755	IGHV2-26
756	IGHV2-5
757	IGHV2-70
758	IGHV3-11
759	IGHV3-13
760	IGHV3-15
761	IGHV3-16
762	IGHV3-20
763	IGHV3-21
764	IGHV3-23
765	IGHV3-30
766	IGHV3-30-3
767	IGHV3-30-5
768	IGHV3-33
769	IGHV3-35
770	IGHV3-38
771	IGHV3-43
772	IGHV3-48
773	IGHV3-49
774	IGHV3-53
775	IGHV3-64
776	IGHV3-66
777	IGHV3-7
778	IGHV3-72
779	IGHV3-73
780	IGHV3-74
781	IGHV3-9
782	IGHV3-38-3
783	IGHV3-69-1
784	IGHV4-28
785	IGHV4-30-1
786	IGHV4-30-2
787	IGHV4-30-4
788	IGHV4-31
789	IGHV4-34
790	IGHV4-39
791	IGHV4-4
792	IGHV4-59
793	IGHV4-61
794	IGHV4-38-2
795	IGHV5-51
796	IGHV5-10-1

797	IGHV6-1
798	IGHV7-4-1
799	IGHV7-81
800	IGK
801	IGKC
802	IGKDEL
803	IGKJ
804	IGKJ1
805	IGKJ2
806	IGKJ3
807	IGKJ4
808	IGKJ5
809	IGKV@
810	IGKV1-12
811	IGKV1-13
812	IGKV1-16
813	IGKV1-17
814	IGKV1-27
815	IGKV1-33
816	IGKV1-37
817	IGKV1-39
818	IGKV1-5
819	IGKV1-6
820	IGKV1-8
821	IGKV1-9
822	IGKV1D-12
823	IGKV1D-13
824	IGKV1D-16
825	IGKV1D-17
826	IGKV1D-33
827	IGKV1D-37
828	IGKV1D-39
829	IGKV1D-42
830	IGKV1D-43
831	IGKV1D-8
832	IGKV2-24
833	IGKV2-28
834	IGKV2-30
835	IGKV2-40
836	IGKV2D-24
837	IGKV2D-28
838	IGKV2D-29
839	IGKV2D-30
840	IGKV2D-40
841	IGKV3-11
842	IGKV3-15
843	IGKV3-20
844	IGKV3-7
845	IGKV3D-11
846	IGKV3D-15
847	IGKV3D-20
848	IGKV3D-7
849	IGKV4-1
850	IGKV5-2
851	IGKV6-21
852	IGKV6D-21
853	IGKV6D-41

854	IGL
855	IGLC1
856	IGLC2
857	IGLC3
858	IGLC6
859	IGLC7
860	IGLJ
861	IGLJ1
862	IGLJ2
863	IGLJ3
864	IGLJ4
865	IGLJ5
866	IGLJ6
867	IGLJ7
868	IGLV@
869	IGLV1-36
870	IGLV1-40
871	IGLV1-44
872	IGLV1-47
873	IGLV1-50
874	IGLV1-51
875	IGLV10-54
876	IGLV11-55
877	IGLV2-11
878	IGLV2-14
879	IGLV2-18
880	IGLV2-23
881	IGLV2-33
882	IGLV2-8
883	IGLV3-1
884	IGLV3-10
885	IGLV3-12
886	IGLV3-16
887	IGLV3-19
888	IGLV3-21
889	IGLV3-22
890	IGLV3-25
891	IGLV3-27
892	IGLV3-32
893	IGLV3-9
894	IGLV4-3
895	IGLV4-60
896	IGLV4-69
897	IGLV5-37
898	IGLV5-39
899	IGLV5-45
900	IGLV5-48
901	IGLV5-52
902	IGLV6-57
903	IGLV7-43
904	IGLV7-46
905	IGLV8-61
906	IGLV9-49
907	C3
908	C5
909	CCL3P1
910	CKLF

911	CMA1
912	CX3CL1
913	CXCL17
914	CCN1
915	EDN1
916	EDN2
917	EDN3
918	FGF10
919	LECT2
920	PPBPP1
921	PROK2
922	SAA1
923	SAA2
924	SBDS
925	SEMA3A
926	SEMA3B
927	SEMA3C
928	SEMA3D
929	SEMA3E
930	SEMA3F
931	SEMA3G
932	SEMA4A
933	SEMA4B
934	SEMA4C
935	SEMA4D
936	SEMA4F
937	SEMA4G
938	SEMA5A
939	SEMA5B
940	SEMA6A
941	SEMA6B
942	SEMA6C
943	SEMA6D
944	SEMA7A
945	SLIT1
946	SLIT2
947	TNC
948	TYMP
949	C5AR1
950	CCR9
951	CCRL2
952	CMKLR1
953	CX3CR1
954	CXCR3
955	CXCR5
956	ACKR3
957	CYSLTR1
958	CYSLTR2
959	ACKR1
960	EDNRA
961	EDNRB
962	FPR1
963	FPR2
964	GPR17
965	GPR32
966	GPR33
967	PTGDR2

968 C5AR2
969 CXCR2
970 LTB4R2
971 PLAUR
972 PLXNA1
973 PLXNA2
974 PLXNA3
975 PLXNA4
976 PLXNB1
977 PLXNB2
978 PLXNB3
979 PLXNC1
980 PLXND1
981 PTAFR
982 ROBO1
983 ROBO2
984 RXFP3
985 XCR1
986 ADM
987 ADM2
988 AGRP
989 AGT
990 AMBN
991 AMELX
992 AMH
993 ANGPTL5
994 ANGPTL7
995 APLN
996 AREG
997 MANF
998 CDNF
999 ARTN
1000 AVP
1001 BDNF
1002 BMP1
1003 BMP10
1004 BMP15
1005 BMP2
1006 BMP3
1007 BMP4
1008 BMP5
1009 BMP6
1010 BMP7
1011 BMP8A
1012 BMP8B
1013 BTC
1014 MYDGF
1015 CALCA
1016 CALCB
1017 CAT
1018 CCK
1019 CD320
1020 CD70
1021 ADA2
1022 CER1
1023 CGA
1024 CGB3

1025	CGB1
1026	CGB2
1027	CGB5
1028	CGB7
1029	CGB8
1030	CHGA
1031	CHGB
1032	CLCF1
1033	CLEC11A
1034	CMTM1
1035	CMTM2
1036	CMTM3
1037	CMTM4
1038	CMTM5
1039	CMTM6
1040	CMTM7
1041	CMTM8
1042	CNTF
1043	CORT
1044	CRH
1045	CSF1
1046	CSF2
1047	CSF3
1048	CSH1
1049	CSH2
1050	CSHL1
1051	CSPG5
1052	CTF1
1053	CCN2
1054	DKK1
1055	EBI3
1056	EGF
1057	EPGN
1058	EPO
1059	EREG
1060	ESM1
1061	FAM3B
1062	FAM3C
1063	FAM3D
1064	FGF1
1065	FGF11
1066	FGF12
1067	FGF13
1068	FGF14
1069	FGF16
1070	FGF17
1071	FGF18
1072	FGF19
1073	FGF20
1074	FGF21
1075	FGF22
1076	FGF23
1077	FGF3
1078	FGF4
1079	FGF5
1080	FGF6
1081	FGF7

1082	FGF8
1083	FGF9
1084	VEGFD
1085	FIGNL2
1086	FLT3LG
1087	FSHB
1088	GAL
1089	GALP
1090	GAST
1091	GCG
1092	GDF1
1093	GDF10
1094	GDF11
1095	GDF2
1096	GDF3
1097	GDF5
1098	GDF6
1099	GDF7
1100	GDF9
1101	GDNF
1102	GH1
1103	GH2
1104	GHRH
1105	GHRL
1106	GIP
1107	GKN1
1108	GMFB
1109	GMFG
1110	GNRH1
1111	GNRH2
1112	GPHA2
1113	GPHB5
1114	GPI
1115	GREM1
1116	GREM2
1117	GRP
1118	GUCA2A
1119	HBEGF
1120	HDGF
1121	HDGFL3
1122	IAPP
1123	IFNE
1124	IFNK
1125	IFNW1
1126	IGF1
1127	IGF2
1128	IL11
1129	IL12A
1130	IL16
1131	IL17B
1132	IL17C
1133	IL17D
1134	IL17F
1135	IL19
1136	IL1F10
1137	IL36RN
1138	IL36A

1139	IL37
1140	IL36B
1141	IL36G
1142	IL1RN
1143	IL20
1144	IL21
1145	IL23A
1146	IL24
1147	IL25
1148	IL26
1149	IFNL3
1150	IL3
1151	IL31
1152	IL32
1153	IL33
1154	IL34
1155	IL5
1156	IL6ST
1157	IL7
1158	IL9
1159	INHA
1160	INHBA
1161	INHBB
1162	INHBC
1163	INHBE
1164	INS
1165	INS-IGF2
1166	INSL3
1167	INSL4
1168	INSL5
1169	INSL6
1170	JAG1
1171	JAG2
1172	FGF7P6
1173	FGF7P3
1174	KITLG
1175	KL
1176	LACRT
1177	LEFTY1
1178	LEFTY2
1179	LHB
1180	LIF
1181	LRSAM1
1182	LTB
1183	LTBP2
1184	LTBP3
1185	LTBP4
1186	MDK
1187	MIA
1188	MLN
1189	MSTN
1190	NAMPT
1191	NDP
1192	NENF
1193	NGF
1194	NMB
1195	NODAL

1196 CCN3
1197 NPFF
1198 NPPA
1199 NPPB
1200 NPPC
1201 NPY
1202 NRG1
1203 NRG2
1204 NRG3
1205 NRG4
1206 NRTN
1207 NTF3
1208 NTF4
1209 NTS
1210 NUDT6
1211 OGN
1212 OSGIN1
1213 OSM
1214 OSTN
1215 OXT
1216 ENDOU
1217 PDGFA
1218 PDGFB
1219 PDGFC
1220 PDGFD
1221 PDGFRL
1222 PGF
1223 PMCH
1224 PNOC
1225 POMC
1226 PPBPP2
1227 PPY
1228 PRL
1229 PRLH
1230 PROK1
1231 PSPN
1232 PTH
1233 PTH2
1234 PTHLH
1235 PTN
1236 PYY
1237 QRFP
1238 RABEP1
1239 RABEP2
1240 REG1A
1241 RETN
1242 RETNLB
1243 RLN1
1244 RLN2
1245 RLN3
1246 SCG2
1247 SCGB3A1
1248 SCT
1249 AIMP1
1250 SECTM1
1251 SLURP1
1252 SPP1

1253 SST
1254 STC1
1255 STC2
1256 TAC1
1257 TDGF1
1258 TDGF1P3
1259 TG
1260 TGFA
1261 TGFB2
1262 TGFB3
1263 THPO
1264 TNFRSF11B
1265 TNFSF12
1266 TNFSF13
1267 TNFSF13B
1268 TNFSF14
1269 TNFSF15
1270 TNFSF18
1271 TNFSF8
1272 TNFSF9
1273 TOR2A
1274 TRH
1275 TSHB
1276 TSLP
1277 TXLNA
1278 UCN
1279 UCN2
1280 UCN3
1281 UTS2
1282 UTS2B
1283 VEGFB
1284 VEGFC
1285 VGF
1286 VIP
1287 ACVR1B
1288 ACVR1C
1289 ACVR2A
1290 ACVR2B
1291 ACVRL1
1292 ADCYAP1R1
1293 ADIPOR1
1294 ADIPOR2
1295 ADRB1
1296 ADRB2
1297 AGTR1
1298 AGTR2
1299 AMHR2
1300 ANGPT1
1301 ANGPT4
1302 ANGPTL1
1303 ANGPTL2
1304 ANGPTL3
1305 ANGPTL4
1306 ANGPTL6
1307 APLNR
1308 AR
1309 AVPR1A

1310 AVPR1B
1311 AVPR2
1312 BMPR1A
1313 BMPR1B
1314 BMPR2
1315 BRD8
1316 C3AR1
1317 CALCR
1318 CALCRL
1319 CNTFR
1320 CRHR1
1321 CRHR2
1322 CRIM1
1323 CRLF1
1324 CRLF2
1325 CRLF3
1326 CSF1R
1327 CSF2RA
1328 CSF2RB
1329 CSF3R
1330 EGFR
1331 ENG
1332 EPOR
1333 ESR1
1334 ESR2
1335 ESRRA
1336 ESRRB
1337 ESRRG
1338 FGFR1
1339 FGFR2
1340 FGFR3
1341 FGFR4
1342 FGFRL1
1343 FLT1
1344 FLT3
1345 FLT4
1346 FSHR
1347 GALR2
1348 GALR3
1349 GCGR
1350 GHR
1351 GHRHR
1352 GHSR
1353 GIPR
1354 GLP1R
1355 GLP2R
1356 GNRHR
1357 GPER1
1358 HNF4A
1359 HNF4G
1360 HTR3A
1361 HTR3B
1362 HTR3C
1363 HTR3D
1364 HTR3E
1365 IFNGR2
1366 IGF1R

1367	IGF2R
1368	IL10RA
1369	IL10RB
1370	IL11RA
1371	IL12RB1
1372	IL12RB2
1373	IL13RA1
1374	IL13RA2
1375	IL15RA
1376	IL2RB
1377	IL17RA
1378	IL17RB
1379	IL17RC
1380	IL17RD
1381	IL17RE
1382	IL18R1
1383	IL18RAP
1384	IL1R1
1385	IL1R2
1386	IL1RAP
1387	IL1RL1
1388	IL1RL2
1389	IL20RA
1390	IL20RB
1391	IL21R
1392	IL22RA1
1393	IL22RA2
1394	IL23R
1395	IL27RA
1396	IL2RA
1397	IL2RG
1398	IL31RA
1399	IL3RA
1400	IL4R
1401	IL5RA
1402	IL6R
1403	IL9R
1404	INSR
1405	KDR
1406	LEPR
1407	LGR4
1408	LGR5
1409	LGR6
1410	LHCGR
1411	LIFR
1412	LTBR
1413	MC1R
1414	MC2R
1415	MC3R
1416	MC4R
1417	MCHR1
1418	MCHR2
1419	MET
1420	MLNR
1421	MPL
1422	MTNR1A
1423	MTNR1B

1424	NGFR
1425	NMBR
1426	NPR1
1427	NPR3
1428	NR0B1
1429	NR0B2
1430	NR1D1
1431	NR1D2
1432	NR1H2
1433	NR1H3
1434	NR1H4
1435	NR1I2
1436	NR1I3
1437	NR2C1
1438	NR2C2
1439	NR2E1
1440	NR2E3
1441	NR2F1
1442	NR2F2
1443	NR2F6
1444	NR3C1
1445	NR3C2
1446	NR4A1
1447	NR4A2
1448	NR4A3
1449	NR5A1
1450	NR5A2
1451	NR6A1
1452	NRP1
1453	NRP2
1454	OGFR
1455	OPRD1
1456	OPRK1
1457	OPRL1
1458	OPRM1
1459	OSMR
1460	OXTR
1461	PGR
1462	PGRMC2
1463	PPARA
1464	PPARD
1465	PRLHR
1466	PRLR
1467	PTGER1
1468	PTGER2
1469	PTGER3
1470	PTGER4
1471	PTGFR
1472	PTH1R
1473	PTH2R
1474	RARA
1475	RARB
1476	RARG
1477	RORA
1478	RORB
1479	RORC
1480	RXFP1

1481	RXFP2
1482	RXRA
1483	RXRB
1484	RXRG
1485	S1PR1
1486	S1PR2
1487	SCTR
1488	SDC1
1489	SDC2
1490	SDC3
1491	SDC4
1492	SORT1
1493	SSTR1
1494	SSTR2
1495	SSTR5
1496	ST2
1497	TACR1
1498	TEK
1499	TGFBR1
1500	TGFBR2
1501	TGFBR3
1502	THRA
1503	THRB
1504	TIE1
1505	TNFRSF10C
1506	TNFRSF10D
1507	TNFRSF11A
1508	TNFRSF12A
1509	TNFRSF13B
1510	TNFRSF13C
1511	TNFRSF14
1512	TNFRSF17
1513	TNFRSF18
1514	TNFRSF19
1515	TNFRSF1A
1516	TNFRSF1B
1517	TNFRSF21
1518	TNFRSF25
1519	TNFRSF4
1520	TNFRSF6B
1521	TNFRSF8
1522	TNFRSF9
1523	TRHR
1524	TSHR
1525	TUBB3
1526	VIPR1
1527	VIPR2
1528	PTPN11
1529	ICAM2
1530	ITGAL
1531	ITGB2
1532	PAK1
1533	NCR2
1534	TYROBP
1535	LCK
1536	FCGR3A
1537	FCGR3B

1538	NCR1
1539	NCR3
1540	CD247
1541	ZAP70
1542	LCP2
1543	LAT
1544	PLCG1
1545	SH3BP2
1546	FYN
1547	SHC2
1548	SHC4
1549	SHC3
1550	SHC1
1551	GRB2
1552	SOS1
1553	SOS2
1554	ARAF
1555	BRAF
1556	RAF1
1557	HCST
1558	CD48
1559	CD244
1560	PRKCA
1561	PRKCG
1562	SH2D1B
1563	SH2D1A
1564	FAS
1565	GZMB
1566	PRF1
1567	CASP3
1568	BID
1569	CD3D
1570	CD3E
1571	CD3G
1572	PTPRC
1573	ITK
1574	TEC
1575	NCK1
1576	NCK2
1577	GRAP2
1578	PAK2
1579	PAK3
1580	PAK4
1581	PAK6
1582	PAK5
1583	RHOA
1584	CDC42
1585	CD28
1586	ICOS
1587	MAP3K8
1588	MAP3K14
1589	CTLA4
1590	CBLC
1591	CBL
1592	CBLB
1593	CDK4
1594	RASGRP1

1595	PDK1
1596	PRKCQ
1597	TRAC
1598	TRAJ1
1599	TRAJ2
1600	TRAJ3
1601	TRAJ4
1602	TRAJ5
1603	TRAJ6
1604	TRAJ7
1605	TRAJ8
1606	TRAJ9
1607	TRAJ10
1608	TRAJ11
1609	TRAJ12
1610	TRAJ13
1611	TRAJ14
1612	TRAJ15
1613	TRAJ16
1614	TRAJ17
1615	TRAJ18
1616	TRAJ19
1617	TRAJ20
1618	TRAJ21
1619	TRAJ22
1620	TRAJ23
1621	TRAJ24
1622	TRAJ25
1623	TRAJ26
1624	TRAJ27
1625	TRAJ28
1626	TRAJ29
1627	TRAJ30
1628	TRAJ31
1629	TRAJ32
1630	TRAJ33
1631	TRAJ34
1632	TRAJ35
1633	TRAJ36
1634	TRAJ37
1635	TRAJ38
1636	TRAJ39
1637	TRAJ40
1638	TRAJ41
1639	TRAJ42
1640	TRAJ43
1641	TRAJ44
1642	TRAJ45
1643	TRAJ46
1644	TRAJ47
1645	TRAJ48
1646	TRAJ49
1647	TRAJ50
1648	TRAJ52
1649	TRAJ53
1650	TRAJ54
1651	TRAJ56

1652	TRAJ57
1653	TRAJ58
1654	TRAJ59
1655	TRAJ61
1656	TRAV1-1
1657	TRAV1-2
1658	TRAV2
1659	TRAV3
1660	TRAV4
1661	TRAV5
1662	TRAV7
1663	TRAV8-1
1664	TRAV8-2
1665	TRAV8-3
1666	TRAV8-4
1667	TRAV8-6
1668	TRAV8-7
1669	TRAV9-1
1670	TRAV9-2
1671	TRAV10
1672	TRAV12-1
1673	TRAV12-2
1674	TRAV12-3
1675	TRAV13-1
1676	TRAV13-2
1677	TRAV14DV4
1678	TRAV16
1679	TRAV17
1680	TRAV18
1681	TRAV19
1682	TRAV20
1683	TRAV21
1684	TRAV22
1685	TRAV23DV6
1686	TRAV24
1687	TRAV25
1688	TRAV26-1
1689	TRAV26-2
1690	TRAV27
1691	TRAV29DV5
1692	TRAV30
1693	TRAV34
1694	TRAV35
1695	TRAV36DV7
1696	TRAV38-1
1697	TRAV38-2DV8
1698	TRAV39
1699	TRAV40
1700	TRAV41
1701	TRBC1
1702	TRBC2
1703	TRBD1
1704	TRBD2
1705	TRBJ1-1
1706	TRBJ1-2
1707	TRBJ1-3
1708	TRBJ1-4

1709	TRBJ1-5
1710	TRBJ1-6
1711	TRBJ2-1
1712	TRBJ2-2
1713	TRBJ2-3
1714	TRBJ2-4
1715	TRBJ2-5
1716	TRBJ2-6
1717	TRBJ2-7
1718	TRBV2
1719	TRBV3-1
1720	TRBV4-1
1721	TRBV4-2
1722	TRBV4-3
1723	TRBV5-1
1724	TRBV5-4
1725	TRBV5-5
1726	TRBV5-6
1727	TRBV5-7
1728	TRBV5-8
1729	TRBV6-1
1730	TRBV6-2
1731	TRBV6-3
1732	TRBV6-4
1733	TRBV6-5
1734	TRBV6-6
1735	TRBV6-7
1736	TRBV6-8
1737	TRBV6-9
1738	TRBV7-2
1739	TRBV7-3
1740	TRBV7-4
1741	TRBV7-6
1742	TRBV7-7
1743	TRBV7-8
1744	TRBV7-9
1745	TRBV9
1746	TRBV10-1
1747	TRBV10-2
1748	TRBV10-3
1749	TRBV11-1
1750	TRBV11-2
1751	TRBV11-3
1752	TRBV12-3
1753	TRBV12-4
1754	TRBV12-5
1755	TRBV13
1756	TRBV14
1757	TRBV15
1758	TRBV16
1759	TRBV17
1760	TRBV18
1761	TRBV19
1762	TRBV20-1
1763	TRBV24-1
1764	TRBV25-1
1765	TRBV27

1766	TRBV28
1767	TRBV29-1
1768	TRBV30
1769	TRDC
1770	TRDD1
1771	TRDD2
1772	TRDD3
1773	TRDJ1
1774	TRDJ2
1775	TRDJ3
1776	TRDJ4
1777	TRDV1
1778	TRDV2
1779	TRDV3
1780	TRGV9
1781	TRGV8
1782	TRGV5
1783	TRGV4
1784	TRGV3
1785	TRGV2
1786	TRGJP2
1787	TRGJP1
1788	TRGJP
1789	TRGJ2
1790	TRGJ1
1791	TRGC2
1792	TRGC1
1793	TRAV6
